# Consonant lengthening marks the beginning of words across a diverse sample of languages

**DOI:** 10.1038/s41562-024-01988-4

**Published:** 2024-09-24

**Authors:** Frederic Blum, Ludger Paschen, Robert Forkel, Susanne Fuchs, Frank Seifart

**Affiliations:** 1 Department of Linguistic and Cultural Evolution, Max-Planck Institute for Evolutionary Anthropology, Leipzig, Germany; 2 Chair for Multilingual Computational Linguistics, University of Passau, Passau, Germany; 3 Leibniz-Zentrum Allgemeine Sprachwissenschaft, Berlin, Germany; 4 Structure et Dynamique des Langues, CNRS, INALCO, IRD, Villejuif, France; 5 Institut für Deutsche Sprache und Linguistik, Humboldt-Universität zu Berlin, Berlin, Germany

**Keywords:** Human behaviour, Language and linguistics

## Abstract

Speech consists of a continuous stream of acoustic signals, yet humans can segment words and other constituents from each other with astonishing precision. The acoustic properties that support this process are not well understood and remain understudied for the vast majority of the world’s languages, in particular regarding their potential variation. Here we report cross-linguistic evidence for the lengthening of word-initial consonants across a typologically diverse sample of 51 languages. Using Bayesian multilevel regression, we find that on average, word-initial consonants are about 13 ms longer than word-medial consonants. The cross-linguistic distribution of the effect indicates that despite individual differences in the phonology of the sampled languages, the lengthening of word-initial consonants is a widespread strategy to mark the onset of words in the continuous acoustic signal of human speech. These findings may be crucial for a better understanding of the incremental processing of speech and speech segmentation.

## Abstract (web summary)

Blum et al. report evidence of lengthening of word-initial consonants across a diverse sample of 51 languages. On average, these consonants are 13 ms longer than word-medial ones, helping mark word boundaries in continuous speech, which is crucial for understanding speech.

## Main

Speech is a continuous stream of acoustic signals that transmit linguistic meaning with the purpose of spoken communication. The intricate process of comprehending speech demands the sequential segmentation of the acoustic signal into discrete units such as words and phrases, which are the basic building blocks of language^1–4^. This segmentation is supported by a complex interaction of factors that operate on the levels of sound structure, lexicon and grammar, both for the speaker and for the listener. Several of these factors have been identified in previous research, but few have been studied across a wide range of languages. Most previous studies on speech production and processing focus on ‘Western, educated, industrial, rich and democratic (WEIRD)’ people and their languages, which undermines the potential to make species-wide generalizations about human language and cognition^5,6^. For the factors that affect speech production, this emerges as a particularly severe limitation in light of the huge variability of grammars and sound systems of the world’s ~7,000 languages^7–9^.

Word onsets play a special role in speech segmentation and word recognition. In the lexicon, word-initial segments are known to be more informative than later segments for distinguishing the intended word from other words^10^, and listeners exploit this for continuously updating hypotheses regarding word identity and boundaries as the phonetic signal progresses^11^. At the level of phonology, word-initial positions generally exhibit more ‘fortition’ (stronger articulation) and fewer ‘lenition’ (weaker articulation) processes than word-internal or word-final positions and are thus assigned a prominent status in phonological theories^12–15^. Complex consonant clusters that are restricted to word onsets through phonotactic constraints may serve as additional cues for word segmentation^16^. However, there is considerable cross-linguistic variation in this respect, and many languages lack consonant clusters altogether. This implies that clusters cannot be a universal method to segment speech into word units. Other, more general strategies may be more relevant instead.

Acoustic features such as modulations of segment duration and changes in fundamental frequency play a major role in structuring speech into different units. Among these features, the lengthening of vowels at the ends of prosodic phrases, clauses or utterances is attested across a wide variety of languages^17,18^ and is often assumed to be universal^19^. At the word level, the acoustic properties of word-initial phones have been argued to be particularly relevant for the prosodic organization of some languages, including English, Korean and French^19–21^. The realization of these word-initial phones may depend on language-specific properties, such as prosodic systems and consonant inventories, but also on between-speaker variation^22,23^. However, so far most of the evidence for these features comes from a handful of languages, most of them Indo-European.

**Fig. 1:**
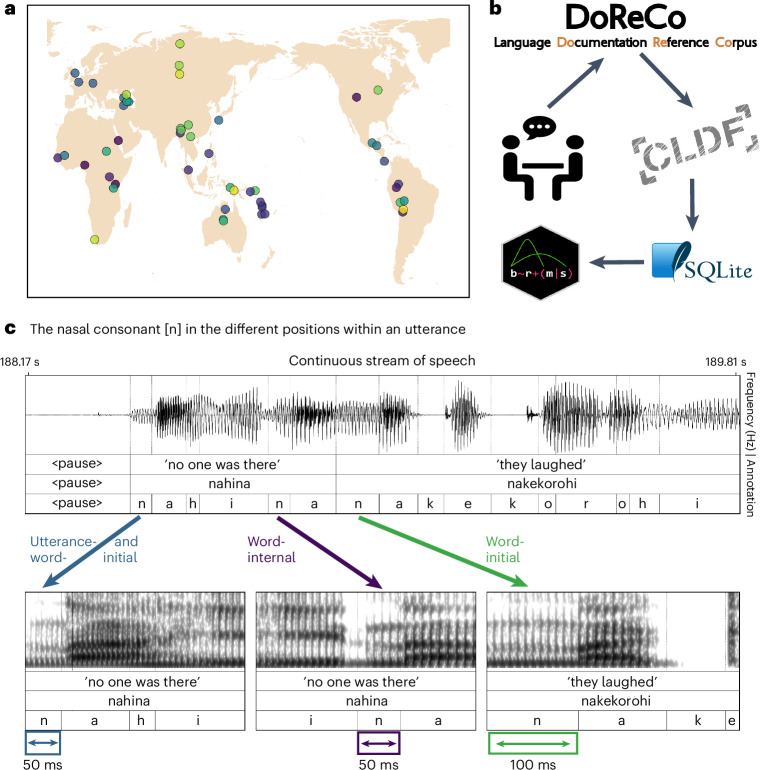
Workflow and language sample. **a**, The geographic distribution of the 51 languages in our sample. The colors indicate the 30 different language families in the sample. **b**, The workflow from fieldwork-based language documentation to the data sample analysed in the present study. **c**, An example (doreco_trin1278_T06, from second 188.17 to 189.81.) of word-initial lengthening in Mojeño Trinitario, an Arawakan language spoken in the Amazonian region of Bolivia^96^.

Two closely related features of word-initial phones that have been reported for individual languages are initial lengthening and strengthening. While initial strengthening implies a stronger articulation^19,23,24^, initial lengthening refers to the duration of consonants. This is illustrated in Fig. 1 from the Amazonian language Mojeño Trinitario, which is also included in our sample. The example illustrates the same consonant /n/ in three different positions: utterance-initial (50 ms), word-internal (50 ms) and word-initial (100 ms). In artificial language learning experiments, it has been shown that speakers of Hungarian, Italian and English can use word-initial consonant lengthening as a cue to locate word boundaries^21^. Similarly, word-initial strengthening has been found to facilitate disambiguation between similar lexical items^25^. However, very little is known about the extent and degree of word-initial lengthening across languages. For words in utterance-initial position, it is not clear whether they display any additional temporal changes. In previous studies, utterance-initial consonants have been found to sometimes be lengthened or shortened, but with an overall small change in duration^26,27^. Indeed, from a functional perspective, it makes sense that no additional cue to word segmentation is necessary at the beginning of utterances, especially after a pause^26,28^. To our knowledge, the cross-linguistic evidence for initial lengthening processes remain scarce, and neither word- nor utterance-initial lengthening has been investigated in a worldwide sample of languages.

Our main research question is whether we can find cross-linguistic evidence for word-initial lengthening or shortening effects in observed speech across a wide range of languages. We also investigate whether we can find such an effect at utterance-initial positions. Following this, we analyse the cross-linguistic distribution of any emergent effects. To be able to make valid generalizations across languages, we also control for between-speaker variability and analyse the lengthening and shortening effects across segments with different places and manners of articulation.

## Results

### Evidence for word-initial lengthening across languages

We used a comprehensive corpus consisting of spontaneous speech from 51 languages, shown in Fig. 1a, recorded from 393 speakers (195 female, 198 male) of an age range between 16 and 100 years^29^. Of these 51 languages, 49 are spoken by non-WEIRD populations^5,6^. The languages in our sample display a wide range of sound inventories and prosodic systems and cover a wide spectrum of grammars. The main units of our analysis are phones (discrete segments of speech); words, as defined by experts on each language; and utterances, which we define as interpausal units—that is, chunks of speech that are not interrupted by a silent pause. The entire corpus consists of over two million phones, all of which have been time-aligned semi-automatically^30^. Of these, we used 874,627 phones for this study (see Methods for information on data filtering). For 49 of 51 languages, our analysis included more than 10,000 data points.

**Fig. 2:**
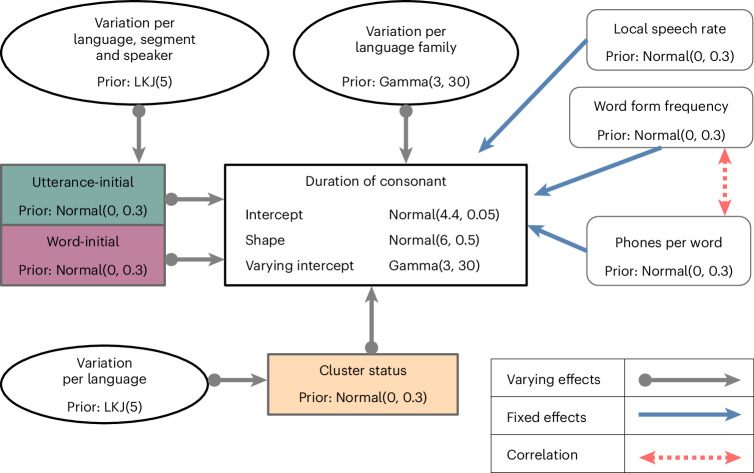
Model architecture. Fixed and varying effects of all parameters in the model including their prior distributions. The prior for the varying slopes is given as Lewandowski-Kurowicka-Joe (LKJ) distribution. The colored boxes indicate the various slopes that were added to the model, varying per language.

We used Bayesian linear regression to estimate the effect of word-initial and utterance-initial positions on the duration of consonants, compared with word-internal positions. We modelled the effect of both positions with a population-level estimate that is allowed to vary between all languages in the sample. For a more conservative analysis, we allowed for variation of the effects between speakers of the same language. This ensures that any inference drawn from the model can be generalized over different speakers. Similarly, we allowed the model to vary between segments of different places and manners of articulation since lengthening effects influence each kind of segment differently^20^. We also controlled for consonant clusters and distinguished between three levels: the consonant is (1) at the beginning of a cluster, (2) in a cluster but not at the beginning or (3) not in a cluster. All levels are modelled as varying between each language. As fixed parameters, we controlled for word length (the number of phones in a word), word form frequency (of forms in the DoReCo corpus of each language) and local speech rate. The full model including prior distributions and likelihood function is given as Fig. 2. The likelihood function defines the response variable using a gamma distribution, which transforms the response variable (duration in milliseconds) to a log scale. Converting to a log scale is a common transformation for duration measures in linguistics to compare orders of magnitude instead of comparing absolute differences in milliseconds^31^. The posterior distributions of parameter values in Bayesian regression studies are defined via their highest posterior density interval (HPDI), which describes the area of the distribution in which most of the sampled posterior values are represented^32–34^. In Bayesian statistics, the type S error rate for the posterior intervals is much lower than in comparable frequentist methods^35^. Another measure to exclude spurious effects and to produce reliable results is to include a region of practical equivalence to 0 (ROPE)^36^. The ROPE is values near 0 (−0.01 to 0.01 on the log scale) that we consider not to be meaningful. In the complete absence of an effect, the posterior distribution would be fully within the ROPE^37^. We interpret 89% HPDIs not overlapping the ROPE as evidence in favour of an effect. If the 89% HPDI overlaps the ROPE, we take the evidence as inconclusive.

**Fig. 3:**
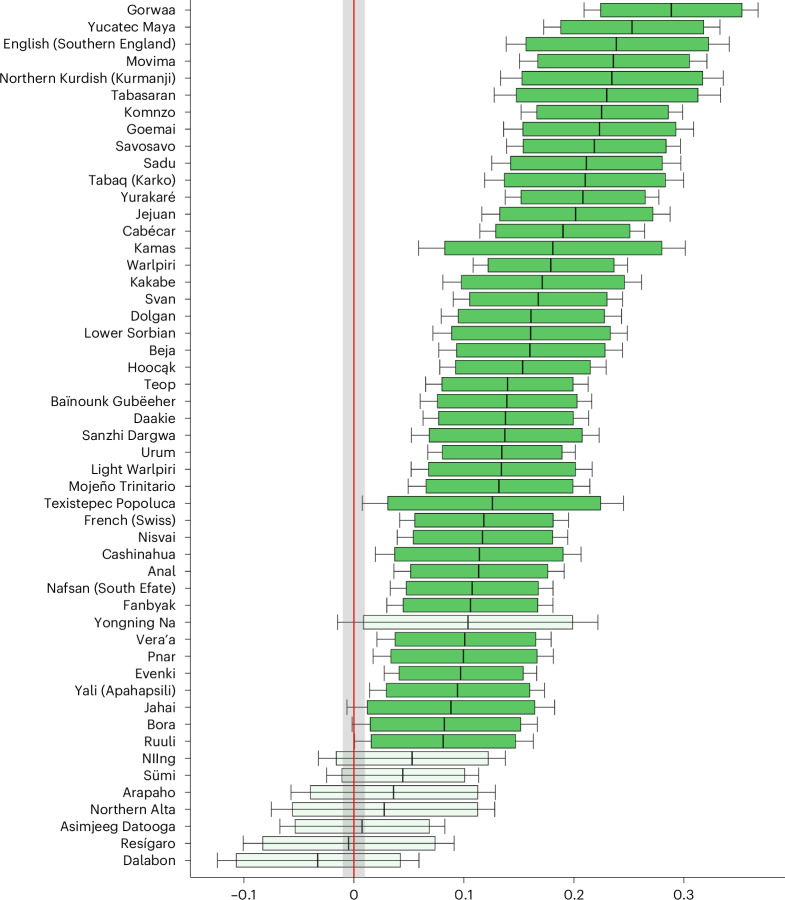
Main results for word-initial lengthening. The value on the x-axis indicates the lengthening effect of the word-initial position on the log-scale. Mean (vertical line), 89% HPDI (box) and 95% HPDI (error bars) (*n* = 6,000 Markov chain Monte Carlo (MCMC) samples) of the posterior distribution for word-initial lengthening across 51 languages. Faded colouring indicates that the 89% posterior interval intersects with the ROPE (grey shading).

The fitted model shows evidence for the word-initial lengthening of consonants in utterance-medial position for 43 of the 51 sampled languages. No language shows evidence in favour of word-initial shortening. For the languages for which we have evidence, the 89% HPDI does not intersect with zero or the values defined in the ROPE. The mean of the HPDI for the 43 languages ranges mostly between 0.1 and 0.3 on the log scale, which translates to an average effect between 8 ms and 18 ms for a segment 84 ms long (the mean duration of phones in the data). The cross-linguistic distribution provides us with high confidence in the reliability of our results. They strongly imply that the observation of lengthening of word-initial consonants in comparison with their word-internal counterparts can be generalized across languages. We show the posterior distributions for the word-initial parameter in all languages in Fig. 3.

**Fig. 4:**
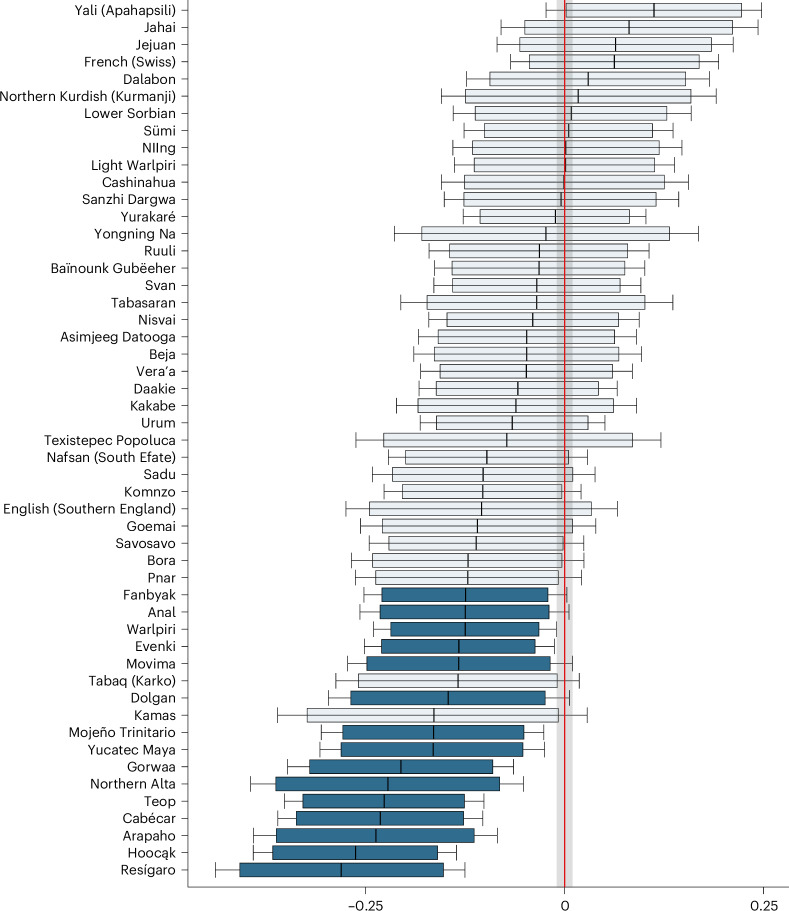
Main results for effects in utterance-initial position. The value on the x-axis indicates the shortening or lengthening effect of the utterance-initial position on the log-scale. Mean (vertical line), 89% HPDI (box) and 95% HPDI (error bars) (*n* = 6,000 MCMC samples) of the posterior distribution for effects in utterance-initial position across 51 languages. Faded colouring indicates that the 89% posterior interval intersects with the ROPE (grey shading).

Regarding utterance-initial positions, no language in our sample shows evidence in favour of lengthening. However, 15 languages show evidence for utterance-initial shortening. In these languages, the duration of consonants tends to be shorter in utterance-initial than in utterance-medial or final position. For the other 36 languages, the results are inconclusive. The HPDI of this distribution displays a weak tendency towards the shortening of utterance-initial consonants for some languages, but for others, the HPDI indicates a weak tendency towards their lengthening. None of those are interpretable, and no uniform cross-linguistic pattern emerges across the sample. We present the individual posterior distributions in Fig. 4.

### Posterior distribution of control variables

**Fig. 5:**
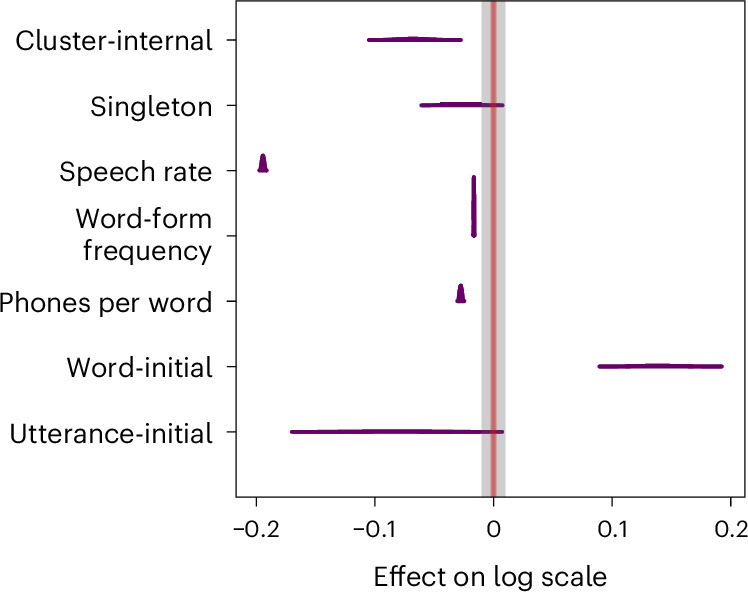
Results for all parameters at the population level. The boxes show the 89% HPDIs (*n* = 6,000 MCMC samples) of the posterior distribution for all parameters at the population level. The point estimates represent variation of less than 0.02 in the estimate of the posterior distribution.

The distribution of parameter values across the whole dataset is presented in Fig. 5. All values are on the log scale. Since the model was parameterized as treatment coding, the ‘non-initial’ level is modelled as the intercept, and both ‘utterance-initial’ and ‘word-initial’ compare directly to the ‘non-initial’ baseline. For the average consonant of 84.35 ms in our data, a lengthening on the log scale of 0.14 (the mean of the word-initial parameter) results in a lengthening of ~13 ms.

Word-form frequency has a small negative effect on duration with a mean of −0.02 (95% HPDI from −0.02 to −0.02) on the log scale. Similarly, word length in phones, measured as phones per word, has a small negative effect on duration with a mean of −0.03 (95% HPDI from −0.03 to −0.03) on the log scale. This is exactly as predicted: segments in longer words are shortened (polysyllabic shortening), and more frequent words are uttered faster. There is a strong correlation (*ρ* = 0.61) between both parameters^31^, in that many phones per word correlates with a lower word-form frequency. Incidentally, this confirms the cross-linguistic validity of Zipf’s law of abbreviation that more frequently used words are shorter^38–41^. Given the strong correlation between both parameters, the effects in the model should not be interpreted separately but should always be considered together statistically. Local speech rate has the expected large effect on duration in the model (−0.19, 95% HPDI from −0.20 to −0.19). As duration per sound is a central part of calculating speech rate, it is not surprising that this predictor is the strongest of all three. It is important to remember that all three predictors are modelled to be uniform across the whole dataset—that is, they are modelled not to vary between individual languages. The effects for cluster-internal consonants show more variation. Consonants outside of a cluster are shorter (−0.03, 95% HPDI from −0.05 to −0.00) than consonants at the beginning of a cluster. Consonants within a cluster are even shorter (−0.07, 95% HPDI from −0.09 to −0.04). The results per language are presented in Supplementary Information section B. Figure 5 further shows that the utterance-initial and word-initial parameters have a large standard deviation at the population level. This indicates that these predictors do not behave uniformly across languages, as we have already seen for the language-specific distributions.

### Posterior evaluation of the model

**Fig. 6:**
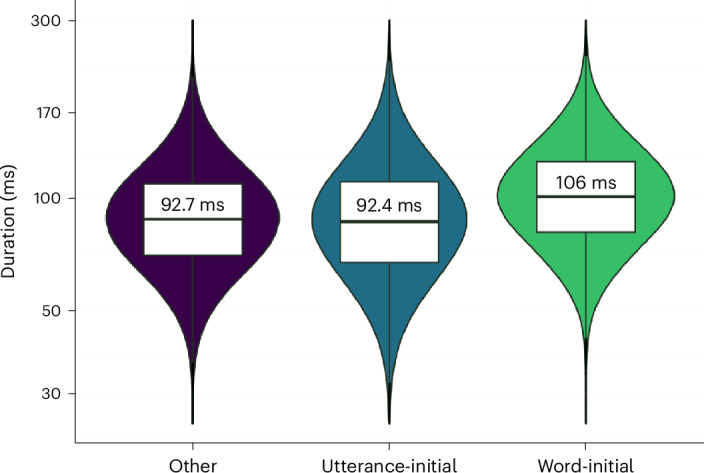
Expected duration across model predictions. Posterior predictions for expected draws (*n* = 6,000 draws from the posterior distribution) given the fitted model and simulated data. The horizontal bar represents the mean with the value printed above it, the box represents the 25th and 75th percentiles, and the violin represents the whole estimated distribution for each parameter. Note that the *y* axis is log scaled.

We ran posterior predictive simulations to confirm that on average, we expect word-initial consonants to be longer than consonants in other positions. A common way to evaluate a Bayesian linear regression model is to run posterior predictions with simulated data^33,34^. We present such posterior predictions in Fig. 6, where we can observe a higher average duration for word-initial consonants than for the other positions. On average, the word-initial consonants in the simulated dataset are expected to be around ~13 ms longer (~106 ms) than consonants in other positions (~93 ms). Full posterior predictive checks according to the Bayesian Analysis Reporting Guidelines^42^ are presented in Supplementary Information section B.

To control for possible non-independence of data points, we carefully analysed the genealogical and spatial relations in our dataset. Our sample includes data from 30 different language families. While eight language families are represented by multiple languages (for example, seven Austronesian, four Indo-European and four Sino-Tibetan languages), there are 22 language families with only one language in our sample. In the model, we added a varying intercept per language family, which shows a very small variance between language families (0.04 on the log scale). This shows that the model cannot identify systematic patterns across language families and attributes most of the durations to variation between languages, segments or speakers. Further approximations of potential correlations between language families are provided by controlling for spatial autocorrelation, since most of the languages in our sample that are related to each other genealogically (especially Austronesian, Indo-European and Sino-Tibetan languages) are also geographically close to each other.

We also verified that the model is not biased through spatial autocorrelation. This type of bias is frequent in linguistic typology and can arise through the borrowing of structural features between languages^43,44^. The amount of spatial autocorrelation in data used for regression models can be measured through the Moran coefficient^45–49^. We based the computation of the Moran coefficient on the geodesic distance between the language coordinates as provided by Glottolog^50^, following suggestions in the literature^51^. We computed this coefficient using the geostan package^46^. In all cases, the coefficient was close to 0, indicating very little or no spatial bias in our data. The full report for each macro area is presented in Supplementary Information section B.

## Discussion

The current study reports acoustic evidence that speakers from vastly different cultural, geographic and linguistic backgrounds produce longer word-initial consonants. While languages differed in the magnitude of lengthening, evidence could be observed across a large part of the sample: 43 languages provided evidence in favour of word-initial lengthening, and none provided evidence for word-initial shortening. The effect in those languages was observed while controlling for the known between-speaker variability in prosodic boundary marking^23^ and the intrinsic differences of lengthening effects of different segments. Since the current study is based on a comprehensive dataset consisting of languages from predominantly non-WEIRD communities from all parts of the world, the distribution of the effect indicates a universal tendency in spoken languages.

Our findings are consistent with models that argue for the dual importance of word-initial lengthening for segmenting speech. First, word-initial lengthening might directly indicate word boundaries. Second, lengthening would facilitate word recognition through the prominent pronunciation of word-initial segments, which are the most informative ones for word identification^10,21^. One potential reason why speakers’ word-initial lengthening is so widespread is that it can promote these two processing requirements for the listener simultaneously^11^. There may be additional articulatory reasons for slowing down in the vicinity of boundaries, but how exactly language comprehension and production interact in this respect remains unclear^28,52^. While the influence of initial lengthening on speech processing has been shown in experimental studies for speakers of some languages^21^, the cross-linguistic evidence for the role of initial lengthening in speech processing would ultimately have to be confirmed in perception studies. Word-initial lengthening could then emerge as an additional key factor for the segmentation of speech in the multi-faceted process of speech recognition^53^.

Regarding speech production, our results partially support and partially contradict predictions made by current models of articulatory phonology, such as the π-gesture model. This model predicts that articulatory gestures are slowed down at prosodic boundaries, manifested in acoustic data as lengthening effects^52,54^. Our findings are, in general, consistent with this view. For larger prosodic boundaries in contrast to smaller ones, the π-gesture model would predict longer durations. If we assume that a word boundary after a pause corresponds to a major prosodic boundary compared with a word boundary with no preceding pause, longer durations should be found in the former than in the latter. However, we did not find a lengthening effect for consonants utterance-initially compared with word-initial positions. For 15 of 51 languages, we even found evidence for shortening of utterance-initial consonants. These findings go against the π-gesture model predictions. The findings do, however, mirror reports on the disappearing effect of final lengthening at strong prosodic boundaries with long pauses^18^. This suggests that speakers systematically modulate the segmental duration of initial consonants at the word level but do not always mark boundaries of higher prosodic levels at the beginning of an utterance. The absence of additional lengthening in utterance-initial position suggests that consonant lengthening is more closely linked to the segmentation and identification of word units than to prosodically structuring speech into larger units such as prosodic phrases. Since utterances are operationalized as chunks of speech surrounded by silent pauses in our study, we interpret the lack of an effect as being related to the lack of functional ambiguity: the first segment following a pause will necessarily also be the first segment of a word, without the need for further segmentation.

Our findings align with several strands of linguistic research about the phonological role of initial segments. At the level of the syllable, onsets have long been recognized as privileged positions. They show several characteristics that other positions do not show, such as resistance to phonological change^15,16,55^. From a diachronic perspective, word-initial consonants tend to be more resistant to phonemic change than consonants in other positions. For example, initial consonant retention is far more typical than initial consonant loss, with some notable exceptions found in Indo-European and across Australian languages^56–58^. Initial consonant deletion as a productive synchronic process is even less common (but see ref. ^59^ for a counterexample). Regarding explanations for such asymmetries, our results lend support to models of evolutionary phonology that view initial strengthening as a cause for the historical development and preservation of ‘strong’ and distinctive word-initial sounds in the phonology and lexicon^60^. There have also been attempts to relate the role of phonological properties to the functional load of syllable onsets compared with syllable codas, and the word-initial position compared with the word-final position^61–63^. One such study investigated the lexical inventories of 12 mostly Indo-European languages and found that syllable onsets have a considerably higher functional load, giving them an extraordinary status^61^. Conversely, word-final positions have been shown to have a reduced degree of structural complexity^63^. These long-term evolutionary processes are consistent with the special role of word-initial segments during the online incremental processing of words.

While the data showed a clear cross-linguistic trend for lengthening at the beginning of words, 8 of 51 languages showed a certain degree of resistance to durational modulations at word-initial position, as evidenced by the intersection of the 89% HPDI with the ROPE (Fig. 3). While this apparent resistance could be explained by insufficient or noisy data, it is also possible that these languages lack word-initial lengthening. Language-specific factors that could affect the degree of lengthening and deserve further attention in future research include the phoneme inventory of the language, the distribution of segments with variable pronunciations (in particular glottal stops), phonological length distinctions (singletons versus geminates) and lexical stress.

Some inevitable limitations might influence the interpretation and generalizability of our findings. First, one limitation of this study lies in the corpus-based approach using aggregated language documentation data and recordings of natural speech. While these data sources provide an ecologically valid and rich set of linguistic samples, they are susceptible to noise and variability inherent in natural speech recordings. They were created over several decades, using different recording equipment and protocols, leading to potential inconsistencies in audio quality. Despite efforts in preselecting high-quality audio for the corpus^30^, the inherent variation in recording conditions remains a concern. However, the corpus-based approach offers the advantage of observing effects in spontaneously produced speech, outside of a strict experimental setting with a less varied sample of texts and speakers.

Second, the sample size, although comprising 51 diverse languages from 30 different language families, still poses a limitation. For some of these languages, we have data from only one (Kamas, Texistepec Popoluca and Yongning Na) or two speakers (Tabasaran, Northern Alta, Kurmanji and Southern British English), while for many other languages, we have data from more than ten speakers. In an ideal scenario, a larger sample size would enhance the study’s generalizability across an even broader spectrum of languages and language families, as well as speakers^64,65^. However, while other multilingual speech corpora are available^66–68^, none of these corpora, in our view, achieve the necessary balance between corpus size, detailed annotation of relevant features and metadata, and expert-informed processing allowing for reliable alignments across a multitude of low-resourced languages that are offered by DoReCo.

A third limitation of the present study lies in its simplistic view of consonant duration. Consonant duration is a multifaceted phenomenon encompassing various acoustic components such as burst, frication, voice onset time and formant transition periods. This also resonates with previous calls for acknowledging the importance of fine phonetic detail for social aspects of communication^69^. Complementing the study with a detailed articulatory perspective that includes annotation of articulatory gestures in the production of consonants could add more depth to our understanding of the underlying principles of word-initial consonant lengthening for specific languages. However, recording and annotating this kind of complex articulatory data is outside the scope of this study. Another limitation related to the previous one is the lack of accounting for word-level prominence in our analysis. Our corpus data are not annotated for suprasegmental features such as stress or tone. However, on the basis of available phonological descriptions, only 4 of the 51 languages can with some certainty be considered to have fixed initial word stress, while most other languages are either tone languages or stress languages with non-initial stress (Supplementary Information section B). In our model, those four languages do not seem to show any patterns for initial-lengthening effects that distinguish them from the other languages. It therefore seems unlikely that our overall results are skewed by not taking word-initial prominence into account.

Despite these limitations, the evidence across a worldwide sample of languages suggests that the lengthening of word-initial consonants is a potentially fundamental process structuring human speech. This strong effect emerges while carefully controlling for between-speaker variability and variability across segments, which adds additional credence to this conclusion. Given the diverse sample of languages in our study, we predict that this effect is replicable for other languages and datasets.

## Methods

### Language sample

Our study uses data from the DoReCo corpus (v.1.2)^29^. The corpus contains time-aligned transcriptions and annotations that mostly originated from language documentation collections covering a wide range of typologically diverse languages. In total, DoReCo v.1.2 contains corpora from 51 languages from 30 language families. All corpora are comparable in size and include at least 10,000 phones (before filtering). A detailed account of the individual corpora and their sources are presented in Extended Data Table 1. Word units in our data were defined and annotated by the language experts who contributed data to DoReCo (Extended Data Table 1), on the basis of current standards in descriptive linguistics. Within DoReCo, the heterogeneous documentation data were processed using a combination of automatic and manual techniques. Forced time alignments were created using the WebMAUS service^70^ first for start and end times of words, which were then corrected manually for the whole corpus^30^. Following this, the updated alignments were used as input to create automatic alignments at the segment level.

We have converted the corpus data to the Cross-Linguistic Data Format (CLDF)^71,72^ to facilitate the reuse of the data and replication of our results. A detailed description of using the corpus as a CLDF dataset is provided as Supplementary Information section A. All preprocessing steps were handled using an SQLite query that is based on the CLDF dataset. Before fitting the models, we cleaned the data by excluding certain observations. Since we are interested only in the lengthening of initial consonants, we removed all vowels from the data. We also removed geminates (that is, phonologically long consonants) due to their intrinsic lengthening. Utterance-initial stops have been excluded because their initial closure period following a pause is unmeasurable^73^. We excluded sounds with a duration equal to or below 30 ms, which was set as the minimum duration by the MAUS aligner, with shorter durations being indicative of imprecise last-resort alignments^30^. Lastly, we excluded outliers beyond three standard deviations of the mean for each speaker. For most speakers, this resulted in an upper threshold of around 300 ms, which is a very conservative threshold concerning the expected duration of individual segments. Random samples of excluded segments showed that these cases are mostly transcription or alignment errors and have been correctly excluded.

### Causal effects on segment duration

In our model, we controlled for several known causal effects on the duration of phones. We controlled for inter- and intra-speaker variation in speech rate through the proxy variable ‘local speech rate’, which is equal to the average duration of phones per utterance. We also controlled for the number of phones per word and the word-form frequency as fixed effects. The word-form frequency is computed as the frequency of each form within the DoReCo corpus core set of each language. Both parameters are predicted to be highly correlated. For frequency of occurrence, more frequent words are known to be shorter (Zipf’s law of abbreviation)^74–77^. Longer words have been shown to have shorter components, most crucially shorter affixes and shorter phones in specific conditions such as under phrasal accent (Menzerath’s law or polysyllabic shortening)^26,39,41,78,79^. In our model, these three variables were log-scaled and standardized for each language.

The effect for word- and utterance-initial position was modelled with varying intercepts and slopes across all languages. This ensured that we could assess the effects in all languages, instead of interpreting the effect on the population level as being true for all languages^80,81^. We also included ‘speaker’ as a varying effect in our model, as there are huge amounts of variation between speakers in all linguistic domains^82–84^. It is necessary to control for this kind of variation to make valid generalizations about language^64,65^. Finally, we controlled for variation of the effect across different segments since there might be variation in the elasticity of segments depending on their place and manner of articulation. In total, the corpus includes 191 different segment types, which are mapped from their X-Sampa representation in DoReCo to the Cross-Linguistic Transcription Systems standard^85,86^.

### Model fitting and evaluation

The reason for choosing a Bayesian approach is the wide range of tools to include prior knowledge of the world in the model and to develop a transparent and reliable model output that is explicit about any uncertainty involved in the inference^87,88^. The goal of our analysis is to determine the effect size of the word-initial position of phones in speech. Given that we know quite a lot about speech sounds in general, such as expected duration and known causal influences, we can add this prior knowledge directly into the model. Bayesian regression offers several well-designed measures for enabling transparency of the workflow^89,90^. We report on all relevant points of the Bayesian Analysis Reporting Guidelines^42^ either in the main text or in the Supplementary Information. We did not include a large-scale sensitivity analysis for our prior distributions, due to the large and energy-intensive computing times. We hope that the prior predictive checks provide sufficient information for the credibility of our prior distributions. We further excluded the points that relate to hypothesis testing with Bayes factors since no model comparison was done in our study. Instead of doing a model comparison or null-hypothesis significance test, we analysed the effect size of our target parameter while controlling for known causal factors.

The model was fit using brms^91,92^, a package in R^93^ that uses cmdstanR as a backend. The model was run with 4,000 MCMC iterations (2,500 for warm-up) on four parallel chains. A computational and visual confirmation of model convergence as well as prior and posterior predictive checks are presented in Supplementary Information section B.

### Reporting summary

Further information on research design is available in the Nature Portfolio Reporting Summary linked to this article.

## Supplementary information

Supplementary Information Supplementary Information sections A and B, including figures.

Reporting Summary

Peer Review File

## Figures and Tables

**Extended Data Table 1:**
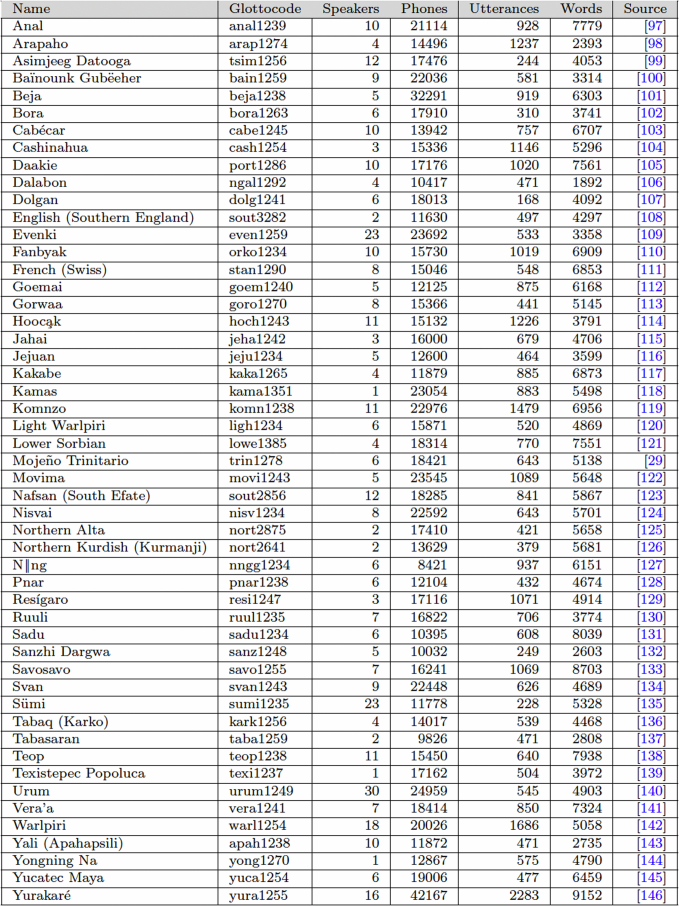
Source Table

Extended Data table with Glottolog language identification code (‘Glottocode’^50^), number of speakers, phones, words, and utterances in our dataset, and source for all languages in the corpus^97–146^.

## Data Availability

For this study, we used data from the DoReCo corpus (v.1.2) and converted them to a CLDF dataset (v.1.2.1)^29,94^. While the data are available as Open Access, some files come with a non-derivative restriction. We have therefore added instructions for an automated workflow of downloading the data and converting it to an SQlite database via CLDF instead of providing the data directly^71,72^, thereby adhering to the non-derivative restrictions. To reproduce the exact steps, please follow the instructions provided in our GitHub repository (https://github.com/FredericBlum/initial_lengthening/blob/v1.0/README.md).

## Code availability

The current version of the code (v.1.0) is available via Zenodo at 10.5281/zenodo.13141902 (ref. ^95^) and curated on GitHub (https://github.com/FredericBlum/initial_lengthening/tree/v1.0). We provide full instructions to reproduce our results in a README.md in the shared repository. The models have been uploaded to an OSF directory (10.17605/OSF.IO/TC9ZX) since we could not upload them to GitHub due to their large file size.
