# Supplementary material for: Consonant lengthening marks the beginning of words across a diverse sample of languages
Source: Nat Hum Behav. 2024 Sep 24;8(11):2127–38. doi: 10.1038/s41562-024-01988-4 (PMC11576513; doi:10.1038/s41562-024-01988-4)
Supplement: Supplementary file 1 — Supplementary Information sections A and B, including figures. [file 41562_2024_1988_MOESM1_ESM.pdf]

nature human behaviour

Supplementary information

https://doi.org/10.1038/s41562-024-01988-4

Consonant lengthening marks the beginning of words across a diverse sample of languages

In the format provided by the authors and unedited

Using the DoReCo CLDF data

Before you use the DoReCo CLDF data you should read through the data model description at cldf/README.md.

Coverage

This dataset is derived from the DoReCo data as follows:

- DoReCo data is **limited to the core data with annotations released under a license without ND (no derivatives) clause** (because we add annotations which would violate this license).
- Morpheme-aligned data is converted into IGT instances in an ExampleTable.
- Two minor issues with the DoReCo data are fixed, namely a handful of phones in the Evenki corpus being linked to two words, and phones/words in the Yucatec corpus having a typo in the speaker reference.
- Links from phones to IPA symbols, and eventually to CLTS sounds are added, based on the orthography profile in etc/orthography.tsv

Note that the `cldfbench.Dataset` implementation in the Python module cldfbench_doreco.py provides functionality to also

- download the ND-licensed data (which is appropriate for analysis, but not for re-destribution)
- download the audio files on which the DoReCo data is based.

To do so,

1. Install the required Python packages (preferably in a fresh virtual environment) via

   ```
   pip install -e .
   ```

2. Download (and unpack) the CLTS v2.2.0 data from DOI: 10.5281/zenodo.5583682.

3. Download (and unpack) the Glottolog v4.8 data from DOI: 10.5281/zenodo.7398962.

4. Then run

   ```
   cldfbench download cldfbench_doreco.py
   ```

   and answer appropriately when prompted.

5. The CLDF data can then be created running

   ```
   cldfbench makecldf cldfbench_doreco.py --glottolog PATH/TO/glottolog-4.8/
   ```

Overview

Due to the size of the DoReCo corpus - ~ 2,000,000 annotated phones (if ND-licensed data is incuded) - analysing the data is made a lot easier (and quicker) when data is accessed via SQL[1] from the CLDF SQLite database.

Create the SQLite database by running

```
cldf createdb cldf/Generic-metadata.json doreco.sqlite
```

An entity relationship diagram, visualizing the schema of the resulting database looks as follows:

ERD

Notes:

- CLDF's `ParameterTable` stores metadata about sounds, linked from `phones.csv`, if `Token_Type` is `xsampa` and an IPA sound corresponding to the X-SAMPA symbol could be determined.
- The database has 4 non-CLDF-standard tables:
  - `glosses.csv`, listing gloss abbreviations used in IGT examples,
  - `speakers.csv`, providing metadata about (core) speakers. Linked from `words.csv`, if available.
  - `words.csv`, listing the time-aligned words in the corpus,
  - `phones.csv`, listing the time-aligned phones in the corpus.

  These non-CLDF-standard tables are named after the corresponding filename. Thus, to prevent the `.` in the name from confusing SQLite, the names must always be quoted, i.e. wrapped in quotes.

Data access via SQL queries

Time-aligned phones and words make up the core contribution of the DoReCo dataset. This data is stored in the `phones.csv` and `words.csv` tables, respectively, and can be queried in a straightforward way.

Retrieving particular phones, e.g. word initials is best done using somewhat advanced SQL constructs like window functions, though.

An SQL query to retrieve these could look as follows:

```sql
SELECT s.* FROM (
    SELECT
        p.*,
        row_number() OVER (PARTITION BY wd_ID ORDER BY cldf_id) rownum
    FROM
        'phones.csv' AS p
    ) AS s
```

[1]For a short overview of SQL and how to access SQL databases (and links to further reading), see https://github.com/dlce-eva/dlce-eva/blob/main/doc/sql.md

```
WHERE
    s.rownum = 1 AND s.token_type = 'xsampa';
```

Now, since SQLite supports views, we can hide this complexity by creating a corresponding view:

```
CREATE VIEW word_initials AS
SELECT s.* FROM (
    SELECT
        p.*,
        row_number() OVER (PARTITION BY wd_ID ORDER BY cldf_id) rownum
    FROM
        'phones.csv' AS p
    ) AS s
WHERE
    s.rownum = 1 AND s.token_type = 'xsampa';
```

and then use this view just like the `phones.csv` table:

```
SELECT COUNT(*) FROM word_initials;
```

Note that views are regular SQL schema objects, and thus persisted in the database, i.e. will survive closing the database connection. Views can be deleted by running a `DROP VIEW <name>` query.

Some useful views are defined in etc/views.sql, and can be "installed" in the database by running

```
sqlite3 -echo doreco.sqlite < etc/views.sql
```

IPA metadata for phones

The default representation for phones in the DoReCo corpus is X-SAMPA. But the CLDF dataset adds IPA representations as well as mappings to CLTS sounds. Thus, information about features of phones can be inferred from the `cldf_cltsReference` column, which stores IDs from https://raw.githubusercontent.com/cldf-clts/clts/v2.2.0/data/sounds.tsv

```
SELECT
  sound_class,
  count(cldf_id) AS word_initial_instances
FROM (
       SELECT
         wi.cldf_id,
          CASE WHEN
            ipa.cldf_cltsReference LIKE '%_consonant' THEN 'consonant'
          ELSE 'vowel' END sound_class
       FROM
         word_initials AS wi,
         parametertable AS ipa
```

```
      WHERE
        wi.cldf_parameterReference = ipa.cldf_id
    ) AS s
GROUP BY sound_class;
```

Which will output a result similar to

sound class	word_initial_instances
consonant	313569
vowel	80820

Utterances

Some kinds of analysis make most sense on utterance level, e.g. computing speech rate. (Utterances are defined as any chunk of speech delimited by silent pauses.) To make this possible, `phones.csv` contains a column `u_id` which allows for aggregating or partitioning phone data by utterance.

Computing speech rate per utterance as *ln(phones per second)* can be done with the following query:

```
SELECT
    p.u_id AS u_id,
    count(p.cldf_id)/sum(p.duration) AS speech_rate
FROM
    'phones.csv' AS p
GROUP BY p.u_id;
```

Again, this could be stored as a view (e.g. `utterances` in etc/views.sql), and joined as needed for speech-rate sensitive analysis.

With a view `utterance_inititials` (see etc/views.sql) we could compute average speech rates per language as follows:

```
SELECT
    w.cldf_languagereference,
    AVG(u.speech_rate) AS sr
FROM
    utterance_initials AS ui,
    'words.csv' AS w,
    utterances AS u
WHERE
    u.u_id = ui.u_id AND ui.wd_id = w.cldf_id
GROUP BY w.cldf_languagereference
ORDER BY sr;
```

After storing this query in a file `sr_by_lang.sql` we can run it and pipe the output to a tool such as termgraph to get a quick overview:

```
$ sqlite3 -csv doreco.sqlite < sr_by_lang.sql | termgraph

kama1351:                8.50
nngg1234:                 9.43
lowe1385:                 9.49
trin1278:                  10.15
yong1270:                  10.21
resi1247:                   10.45
sadu1234:                   10.54
arap1274:                   10.92
tsim1256:                    11.40
even1259:                    11.56
sanz1248:                    11.57
beja1238:                    11.58
orko1234:                     11.59
jeha1242:                     11.62
bora1263:                     11.72
sout3282:                     11.76
svan1243:                     11.86
dolg1241:                      11.89
goem1240:                      11.97
movi1243:                      12.03
cash1254:                      12.05
anal1239:                       12.18
kaka1265:                       12.20
stan1290:                       12.30
teop1238:                       12.35
taba1259:                       12.41
kark1256:                        12.49
pnar1238:                        12.54
ngal1292:                         12.78
sout2856:                         12.84
ruul1235:                         12.99
komn1238:                         13.03
jeju1234:                          13.08
savo1255:                          13.12
port1286:                          13.30
sumi1235:                           13.43
apah1238:                            13.81
goro1270:                             14.08
nort2641:                             14.11
bain1259:                              14.27
texi1237:                              14.41
vera1241:                                14.85
```

Speaker information

Speaker information is available from the `speakers.csv` table, which can be joined to phones via `words.csv`. E.g. the following query counts phones per speaker sex.

```
SELECT
    s.sex AS sex,
    count(p.cldf_id) AS num_phones
FROM
    'phones.csv' as p,
    'words.csv' as w,
    'speakers.csv' as s
WHERE
    p.wd_id = w.cldf_id AND w.speaker_id = s.cldf_id
GROUP BY s.sex;
```

sex	num_phones
f	721358
m	1142344

Correlations between speaker metadata and IPA phone metadata can be assessed by joining the ParameterTable as well

```
SELECT
    s.sex AS sex,
    ipa.cldf_name AS IPA,
    count(p.cldf_id) AS num
FROM
    'phones.csv' AS p,
    'words.csv' AS w,
    'speakers.csv' AS s,
    parametertable AS ipa
WHERE
    p.wd_id = w.cldf_id AND
    w.speaker_id = s.cldf_id AND
    p.cldf_parameterReference = ipa.cldf_id AND
    ipa.cldf_cltsreference LIKE '%breathy%'
GROUP BY p.cldf_name, s.sex;
```

lending superficial support to the hypothesis of "breathiness as a feminine voice characteristic" (DOI: 10.1016/j.jvoice.2007.08.002):

sex	IPA	num
f	l	31

sex	IPA	num
m	l	4
f	n	16
m	n	7

IGT examples

Although the main focus of the DoReCo dataset are the time-aligned phones, the data also contains many glossed example sentences. These are available in the CLDF ExampleTable.

So, for example, if we are interested in particular phenomena, we can first inspect the gloss labels used for a language, and the number of examples containing the label:

```
SELECT
    g.cldf_name AS label,
    count(DISTINCT e.cldf_id) AS freq
FROM
    'glosses.csv' AS g,
    exampletable AS e
WHERE
    e.cldf_gloss LIKE '%' || g.cldf_name || '%' AND
    g.cldf_languagereference = 'sout3282' AND
    e.cldf_languagereference = 'sout3282'
GROUP BY g.cldf_name
ORDER BY freq
LIMIT 3;
```

Notes:

- We use SQLite's string concatenation operator `||` to string together a suitable argument for the `LIKE` operator, making sure we match gloss containing `HORT`.
- To make sure the `LIKE` operator matches in a case sensitive way, you may have to issue a `PRAGMA` statement to force the correct behaviour:

  ```
  sqlite> PRAGMA case_sensitive_like=ON;
  ```

label	freq
SUBJ	1
HORT	3
SUPR	7

And now we can list the aligned analyzed words and gloss lines of the matching IGT examples:

```sql
SELECT
    cldf_analyzedword || char(10) || cldf_gloss || char(10)
FROM
    exampletable
WHERE
    cldf_gloss LIKE '%HORT%' AND cldf_languagereference = 'sout3282' ;
```

Note: We use SQLite's `char` function to insert newline characters in the output to "align" the IGTs.

```
****    and he  says    ****    put tha'    pony    in  ****
****    and 3SG.M   say.PRS.3SG ****    put.IMP DIST.SG pony    in  ****

he  says    and ****    in  the cart    ****    and ****
3SG.M   say.PRS.3SG and ****    in  the cart    ****    and ****

let's   try him ****    he  says    ****    I   want    that
HORT    try.INF 3SG.M.OBL   ****    3SG.M   say.PRS.3SG ****    1SG want.PRS    DIST.SG

for Tom-Smith   atFaversham ****    if  it  suit-s  him
for Tom-Smith   at  Faversham   ****    if  3SG.N   suit-PRS.3SG    3SG.M.OBL

****    well    ****    put him in  ****    let's   try him
****    well    ****    put.IMP 3SG.M.OBL   in  ****    HORT    try.INF 3SG.M.OBL

[INT]   ****    let's   leave   that    for another occasion    shall-we
[INT]   ****    HORT    leave.INF   DIST.SG for another occasion    shall-2PL
```

Audio files

The DoReCo CLDF dataset also includes information about the publicly available audio files underlying the corpora. The `start` and `end` columns in `words.csv` and `phones.csv` provide time stamps relative to the linked files.

Thus, the following query retrieves the longest word in the sout3282 corpus, and its position in the audio file:

```sql
sqlite>
    SELECT
        w.cldf_id,
        w.cldf_exampleReference,
        w.cldf_name,
        count(p.cldf_id) AS wl,
        f.cldf_downloadUrl,
        w.start,
        w.end
    FROM
        'phones.csv' AS p,
```

```
        'words.csv' AS w,
        mediatable AS f
    WHERE
        p.wd_id = w.cldf_id AND
        w.cldf_languageReference = 'sout3282' AND
        w.cldf_mediaReference = f.cldf_id
    GROUP BY w.cldf_id
    ORDER BY wl DESC
    LIMIT 1;
sout3282_w020797|sout3282-1322|agricultural|12|
https://api.nakala.fr/data/10.34847%2Fnkl.dcfbh2yw/
830afcf32230aa859b39a92137edbdb18c5b174f|
1950.493|1951.383
```

Plugging this data into HTML as follows

```
<html>
<body>
<p>agricultural</p>
<audio preload="auto"
       controls="controls"
       src="https://api.nakala.fr/data/10.34847%2Fnkl.dcfbh2yw/
       830afcf32230aa859b39a92137edbdb18c5b174f#t=1950.493,1951.383">
</audio>
</body>
</html>
```

and saving the file as `agricultural.html` you can get a light-weight corpus viewer by opening the file in your browser and clicking the play button to listen to the word.

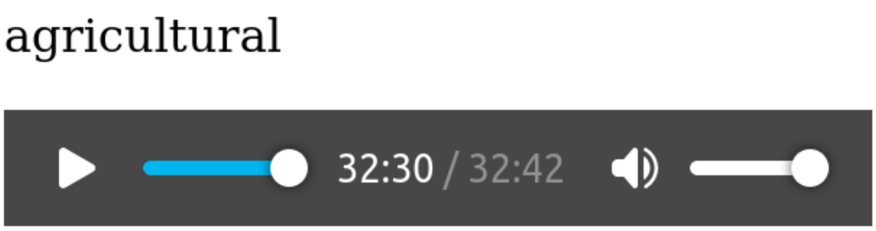

Figure 1: audio player

IGT examples are linked to audio files as well. So we can do the same for the glossed example linked to the word *agricultural*:

```
SELECT
```

```
    e.cldf_primaryText,
    '<table><tr>' || char(10) || '<td>' ||
      replace(e.cldf_analyzedword, char(9), '</td><td>') || '</td></tr>' || char(10) ||
       '<tr><td>' ||  replace(e.cldf_gloss, char(9), '</td><td>') || '</td></tr></table>',
    e.cldf_translatedText,
    f.cldf_downloadUrl,
    e.start,
    e.end
FROM
    exampletable AS e,
    mediatable AS f
WHERE
    e.cldf_mediareference = f.cldf_id AND e.cldf_id = 'sout3282-1322';
```

Note: While somewhat cumbersome, basic HTML can be strung together in SQL. In the above code we use the SQLite operator `||` to concatenate strings, and the char function to express the "special" characters `\n` - newline - and `\t` - tab, and the replace function to turn the tab-separated aligned words into HTML table cells.

Going further

Parametrized queries

To make it easier to run parametrized queries (aka queries with placeholders), this dataset - when installed via `pip install -e .` - registers a cldfbench command `query`. So, with a query

```
SELECT count(*) from `phones.csv` where duration > ?;
```

in a file called `phones_by_duration.sql` you can run the parametrized query from the commandline:

```
$ cldfbench doreco.query phones_by_duration.sql 0.7
  count(*)
----------
     79281
```

Filtering phones based on features

Since phones are mapped to BIPA sounds listed in `ParameterTable`, they can be filtered based on feature values, because the column `ParameterTable.cldf_cltsReference` contains the sound name, which is a _ delimited concatenation of feature values for the sound.

Thus, for example selecting pulmonic consonants can be done with SQL as follows

```
SELECT * FROM
    `phones.csv` AS phone,
```

```
    parametertable as sound
WHERE
    phone.cldf_parameterReference = sound.cldf_id AND
    sound.cldf_cltsReference LIKE '%_consonant' AND
    sound.cldf_cltsReference NOT LIKE '%click%' AND
    sound.cldf_cltsReference NOT LIKE '%implosive%' AND
    sound.cldf_cltsReference NOT LIKE '%ejective%'
;
```

Filtering outliers

If you are accessing the DoReCo SQLite data from R, you can make use of math extensions available with RSQLite to push the "heavy lifting" when filtering outliers down to the database.

Thus, filtering out phones that are unusually long for the respective speaker (indicating incorrect annotation) can be done running SQL as follows

```
> sql = "SELECT
    count(*)
FROM
    `phones.csv` AS phone,
    `words.csv` AS word
LEFT JOIN
    (
        SELECT
            w.speaker_id, avg(p.duration) + 3 * stdev(p.duration) AS threshold
        FROM
            `phones.csv` AS p,
            `words.csv` AS w
        WHERE
            p.cldf_parameterreference IS NOT NULL AND
            p.wd_id = w.cldf_id
        GROUP BY w.speaker_id
    ) AS t
ON
    word.speaker_id = t.speaker_id
WHERE
    phone.cldf_parameterReference IS NOT NULL AND
    phone.wd_id = word.cldf_id AND
    phone.duration < t.threshold
;"
```

via RSQLite:

```
> library(DBI)
> library(RSQLite)
> db <- dbConnect(RSQLite::SQLite(), "doreco.sqlite")
```

```
> RSQLite::initExtension(db)
> dbGetQuery(db, sql)
count(*)
1  1681433
```

Note that the SQLite library used in `cldfbench doreco.query` does also support math functions as well as `stdev` as aggregate function. Thus, the above query can also be run by saving the SQL as `query,sql` and running

```
cldfbench doreco.query q.sql
  count(*)
----------
   1681433
```

Supplementary B: Model Explorations

July 29, 2024

1 Explorative analysis of the raw data

The DoReCo data has some peculiarities which need to be discussed. While the word boundaries have been corrected by hand, the time-alignment of the individual phones has been done via WebMAUS [19]. This leads to two very relevant characteristics of the phoneme-data: a) The phones in the data have a minimum value of 30ms, and b) WebMaus has, just as humans, a preference for round numbers. The minimum value of 30ms leads to a pooling of all reduced/elided phones to that value. In case a corpus creator has annotated a phone because it 'belongs there', but it's not actually spoken, it will receive the minimum value. If a phone is very reduced, the same occurs. This means that all phones at this value are no reliable estimates, which is why we removed them from the dataset. The preference for round numbers can be observed from Fig. 1. The distribution of the data peaks at 40ms, 50ms, ff., with only few phones in between.

We can also read two other important characteristics of the plot: i) There are a lot more non-initial consonants than word- or utterance-initial consonants, and ii) the mean duration of utterance- (87.2ms) and word-initial consonants (88.7ms) is slightly higher than that of non-initial consonants (82.5). The grand average phone duration in the filtered dataset is 84.3ms.

2 Prior Predictive Checks

A visual representation of the prior distribution for all parameters is given in Figure 2. All priors are weekly informative, based on expected duration of phonemic segments in speech (between 50ms and 150ms), and magnitude of expected parameter values. The target parameters for our analysis (word- and utterance-initial) are not biased towards any directions, being defined through a normal prior distribution with mean 0.

Simulating data based on the prior values results in data samples that are similar to the observed data, but span a wider distribution. This is exemplified in Figure 3, where five (top) and ten (bottom) data simulations are compared to the observed data.

3 Model Convergence

The visual evaluation with trace and trunk plots of the Markov Chain sampling in Figure 4 confirms some noisiness of the intercept estimation, but also the fact that all four chains sample the same space [37]. The noisiness is likely result of the fact that we account for varying intercepts of 393 speakers as well as 191 segments in all different languages. This is a lot of variation to estimate, and the noise comes to no surprise of us.

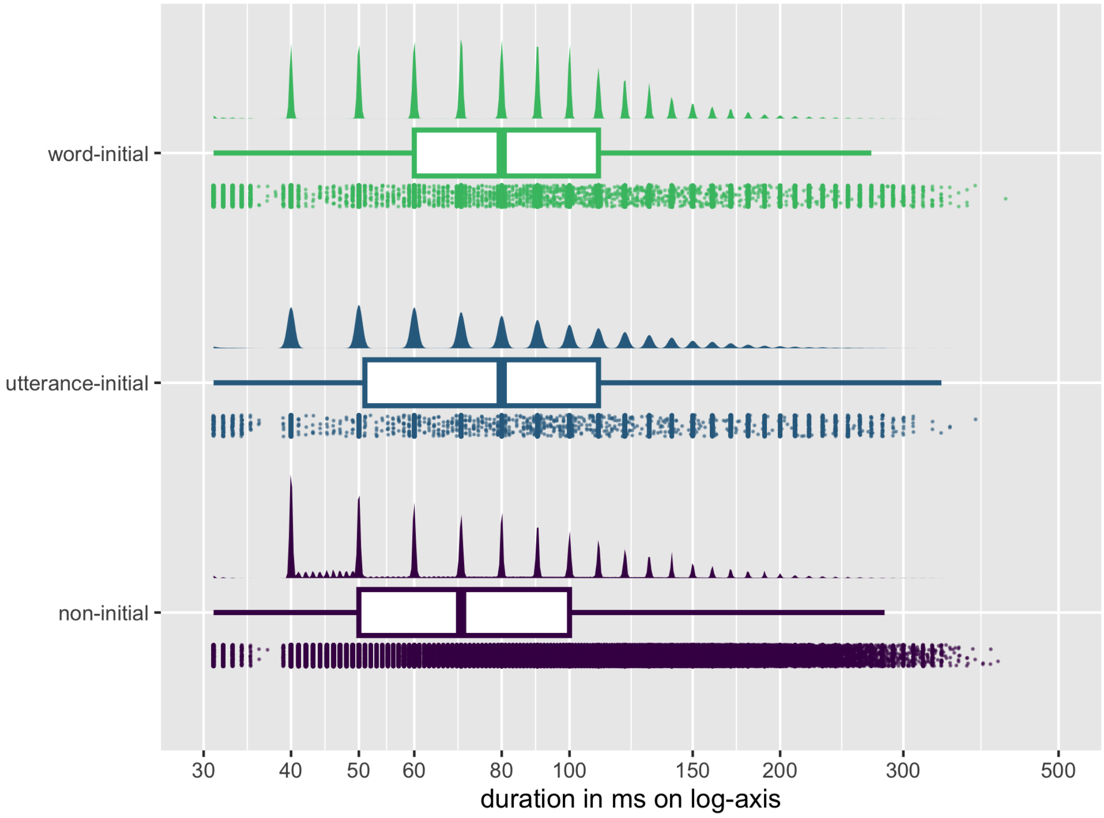

Figure 1: Density distribution for phones across positions.

For a quantitative convergence check, we use two different measures. All $\hat{R}$-values are below 1.01. The ESS is low for some of the varying intercepts, which is not too surprising considering the low sample size for some of the speakers and segments. The amount of phones per speakers varies widely, and as a result some speakers may have a very low amount of samples of certain categories, leaving more work to do for the model to estimate. The overall estimation of the posterior probabilities is only barely affected by this.

4 Posterior Predictive Checks

We use the posterior predictive checks as implemented in the bayesplot package [10, 11]. As Figure 6 shows, the simulated data based on the posterior distributions of the fitted model has similar distributions as the original data, both for the ungrouped (Figure 6A) and the grouped (Figure 6B) overlay.

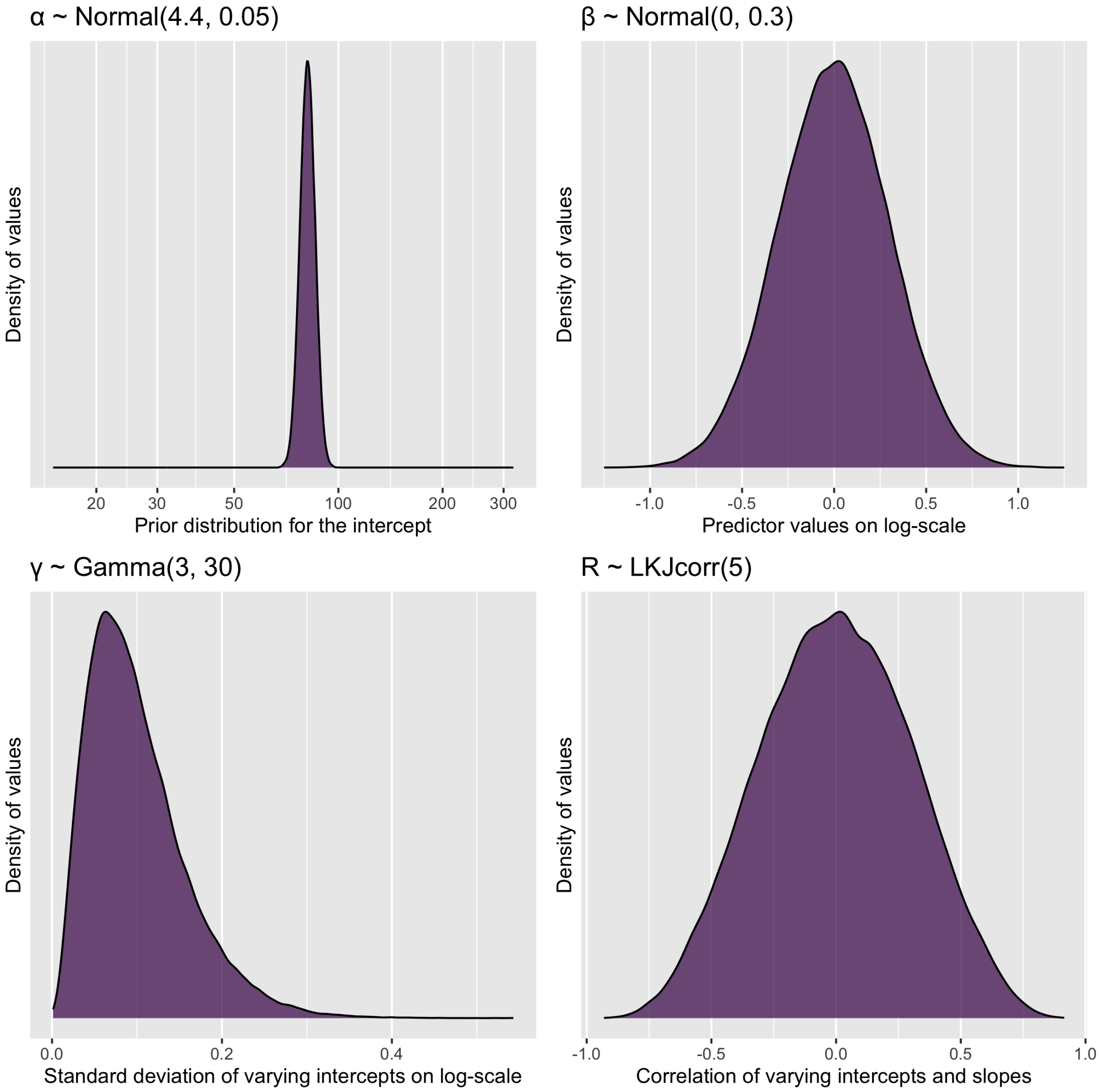

Figure 2: Prior distribution for all population-level parameters.

5 Consonants in clusters across languages

Figure 7 shows the posterior distribution for the control variable consonant clusters, as suggested by an anonymous reviewer. The languages show some variation and some even go against the commonly assumed patterns and have longer consonants in the middle of consonant clusters than at their beginning (Savosavo, Jahai). The reason for this are probably language-specific phonotactics.

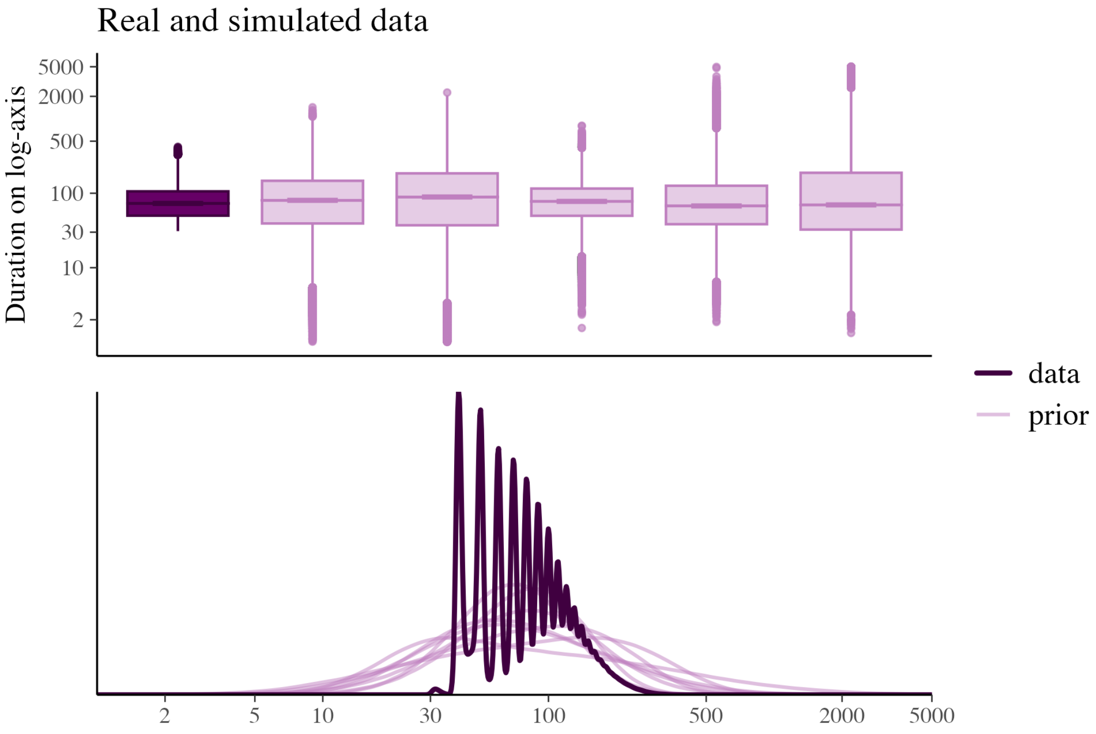

Figure 3: Data simulation based on prior distributions.

6 Word-initial lengthening across speakers and sound classes

The effect is consistent across all speakers of each language. As we show in Fig. 8, there is not a single speaker with word-initial shortening of consonants. This provides further arguments for the potential universality of the effect. For segments, no clear patterns emerge. In both word- and utterance-initial position, some segments are shortened and some are lengthened. For most consonants however, the evidence is not conclusive.

7 Moran coefficient for macro-areas

This section presents the Moran coefficient in our data for all macro areas. The results are all close to 0, which indicates little to no spatial auto-correlation.

8 Accent in the languages of the sample

Name	Glottocode	Type	Source
Anal	anal1239	tone language	[26]
Arapaho	arap1274	tone language	[5]
Asimjeeg Datooga	tsim1256	tone language	[13]
Baïnounk Gubëeher	bain1259	unclear, not documented	
Beja	beja1238	stress language, lexical	[36]
Bora	bora1263	tone language	[34]
Cabécar	cabe1245	unclear, pitch accent (?)	[38]
Cashinahua	cash1254	unclear, not documented	
Daakie	port1286	unclear, not documented	
Dalabon	ngal1292	unclear, not documented	
Dolgan	dolg1241	unclear, not documented	
English (Southern England)	sout3282	stress language, lexical	[6]
Evenki	even1259	stress language, lexical	[25]
Fanbyak	orko1234	unclear, not documented	
French (Swiss)	stan1290	phrase language	[8]
Goemai	goem1240	tone language	[17]
Gorwaa	goro1270	unclear, pitch accent (?)	[15]
Hoocąk	hoch1243	stress language, third-from-left	[14]
Jahai	jeha1242	stress language, final	[3]
Jejuan	jeju1234	phrase language	[43]
Kakabe	kaka1265	tone language	[39]
Kamas	kama1351	unclear, conflicting accounts	[20]
Komnzo	komn1238	stress language, initial	[7]
Light Warlpiri	ligh1234	unclear, not documented	
Lower Sorbian	lowe1385	stress language, initial	[29]
Mojeño Trinitario	trin1278	stress language, penultimate	[28]
Movima	movi1243	stress language, penultimate	[16]
Nafsan (South Efate)	sout2856	stress language, initial	[33]
Nisvai	nisv1234	unclear, not documented	
Northern Alta	nort2875	stress language, lexical	[12]
Northern Kurdish (Kurmanji)	nort2641	stress language, final	[32]
N‖ng	nngg1234	tone language	[4]
Pnar	pnar1238	stress language, final	[27]
Resígaro	resi1247	tone language	[2]
Ruuli	ruul1235	tone language	[23]
Sadu	sadu1234	tone language	[42]
Sanzhi Dargwa	sanz1248	stress language, lexical	[9]
Savosavo	savo1255	stress language, penultimate	[40]
Svan	svan1243	unclear, not documented	
Sümi	sumi1235	tone language	[31]
Tabaq (Karko)	kark1256	tone language	[18]
Tabasaran	taba1259	stress language, lexical	[1]
Teop	teop1238	unclear, not documented	
Texistepec Popoluca	texi1237	stress language, quantity-based	[41]
Urum	urum1249	unclear, not documented	
Vera'a	vera1241	stress language, lexical	[30]
Warlpiri	warl1254	stress language, initial	[24]
Yali (Apahapsili)	apah1238	unclear, not documented	
Yongning Na	yong1270	tone language	[22]
Yucatec Maya	yuca1254	tone language	[21]
Yurakaré	yura1255	stress language, (pen)ultimate	[35]

Table 1: Broad characterization of word prosody types found in the languages in our sample. The table indicates whether a language can be classified as a stress language, a tone language, or a phrase language (cf. [8]), and in case of stress languages, whether stress is lexical, quantity-based, or fixed to a certain position. For several languages, to our knowledge, no reliable documentation of their word prosody exists to date.

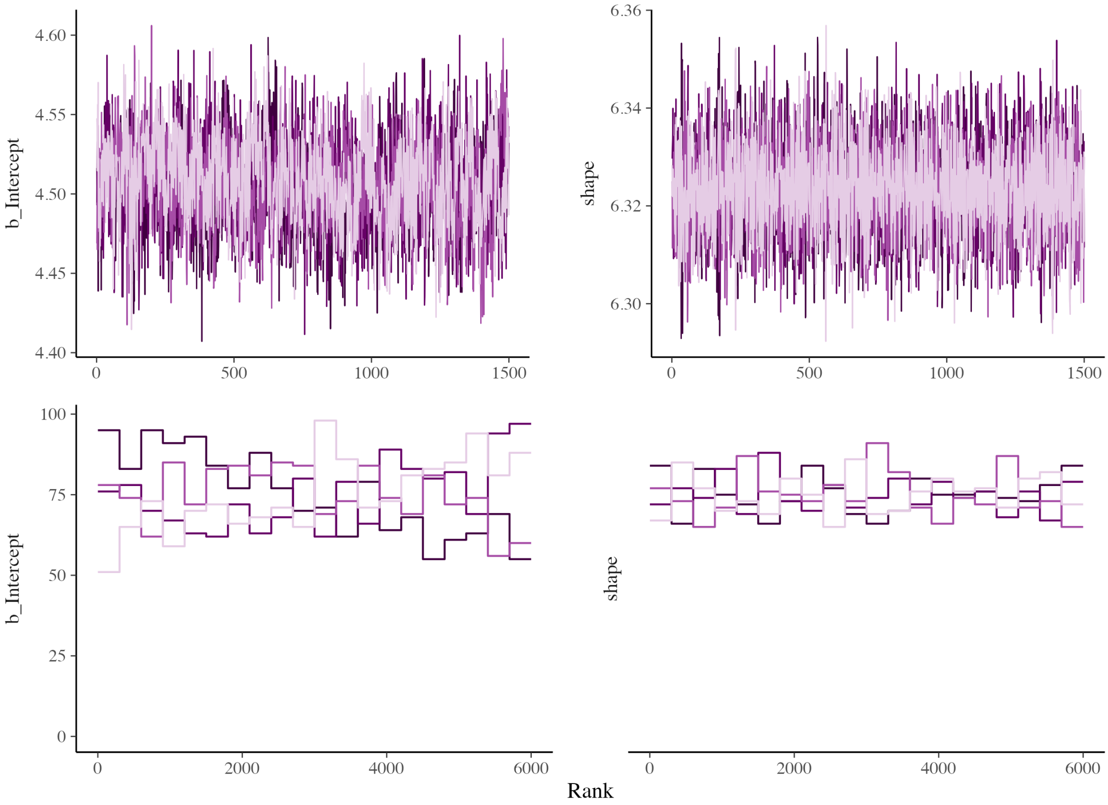

Figure 4: Visual evaluation of the MCMC sampling with trace (top) and trunk (bottom) plots.

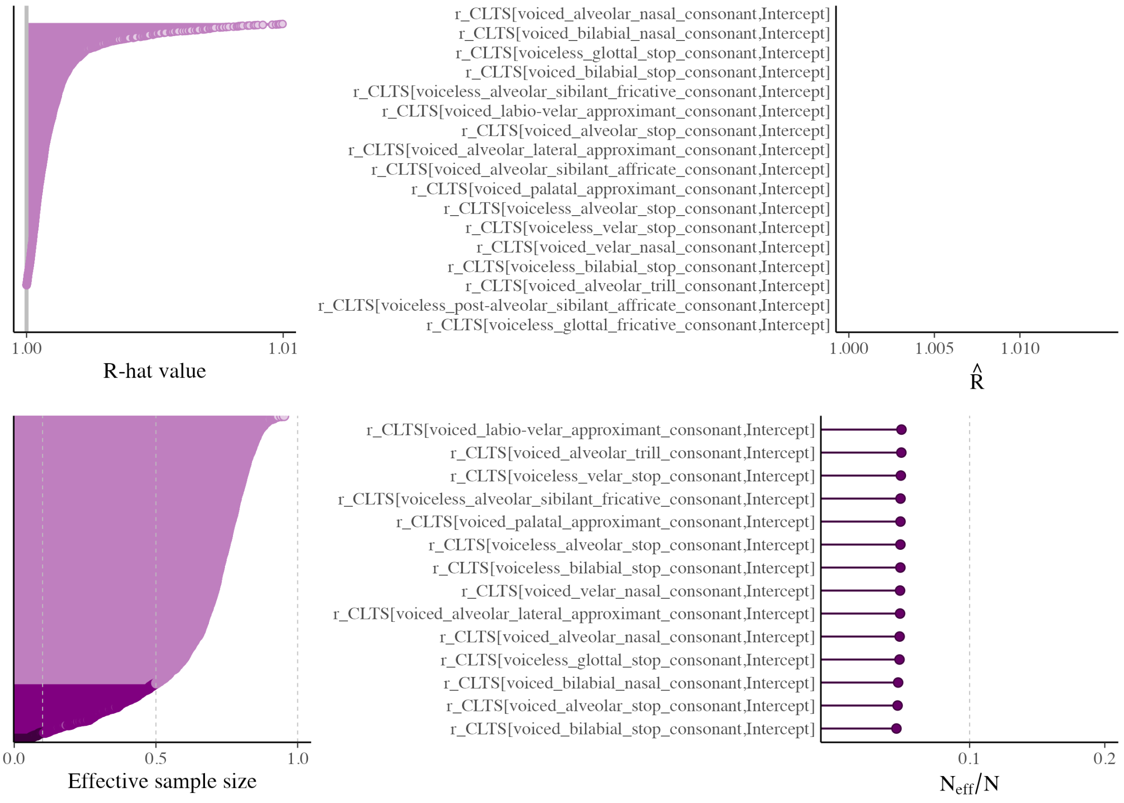

Figure 5: $\hat{R}$ and Effective Sample Size for all parameters (left) and the parameters with highest (for $\hat{R}$) and lowest (for ESS) values.

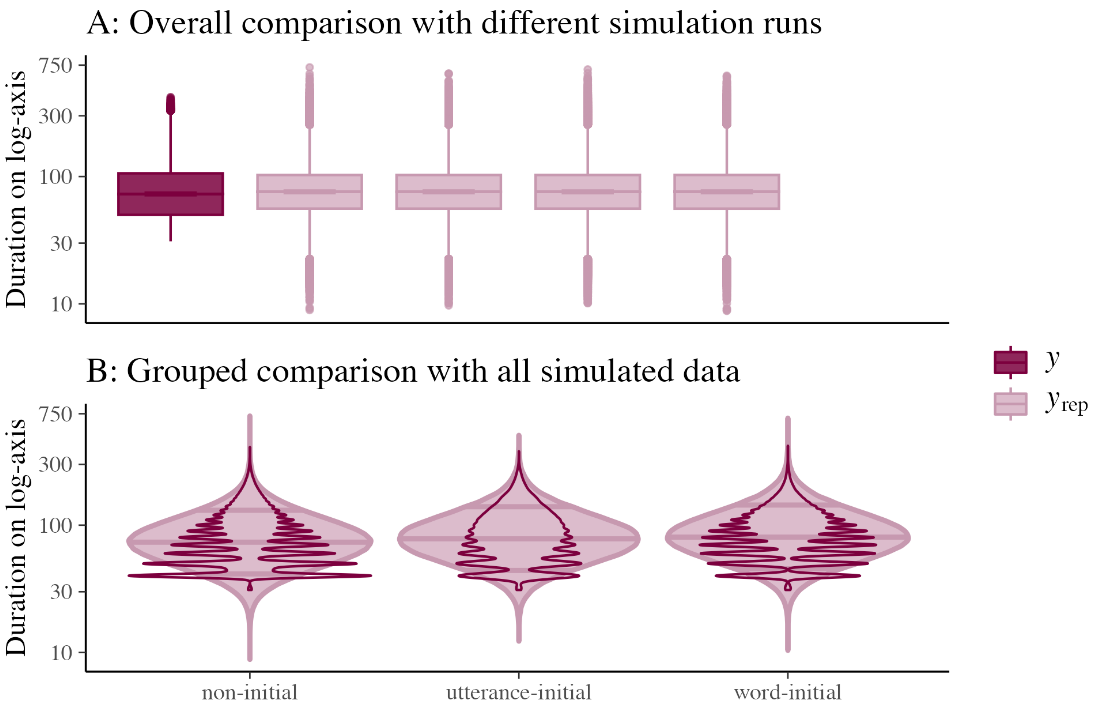

Figure 6: The Posterior Predictive Check for simulated data based on the parameter distribution of the fitted model shows the adequate sampling of data.

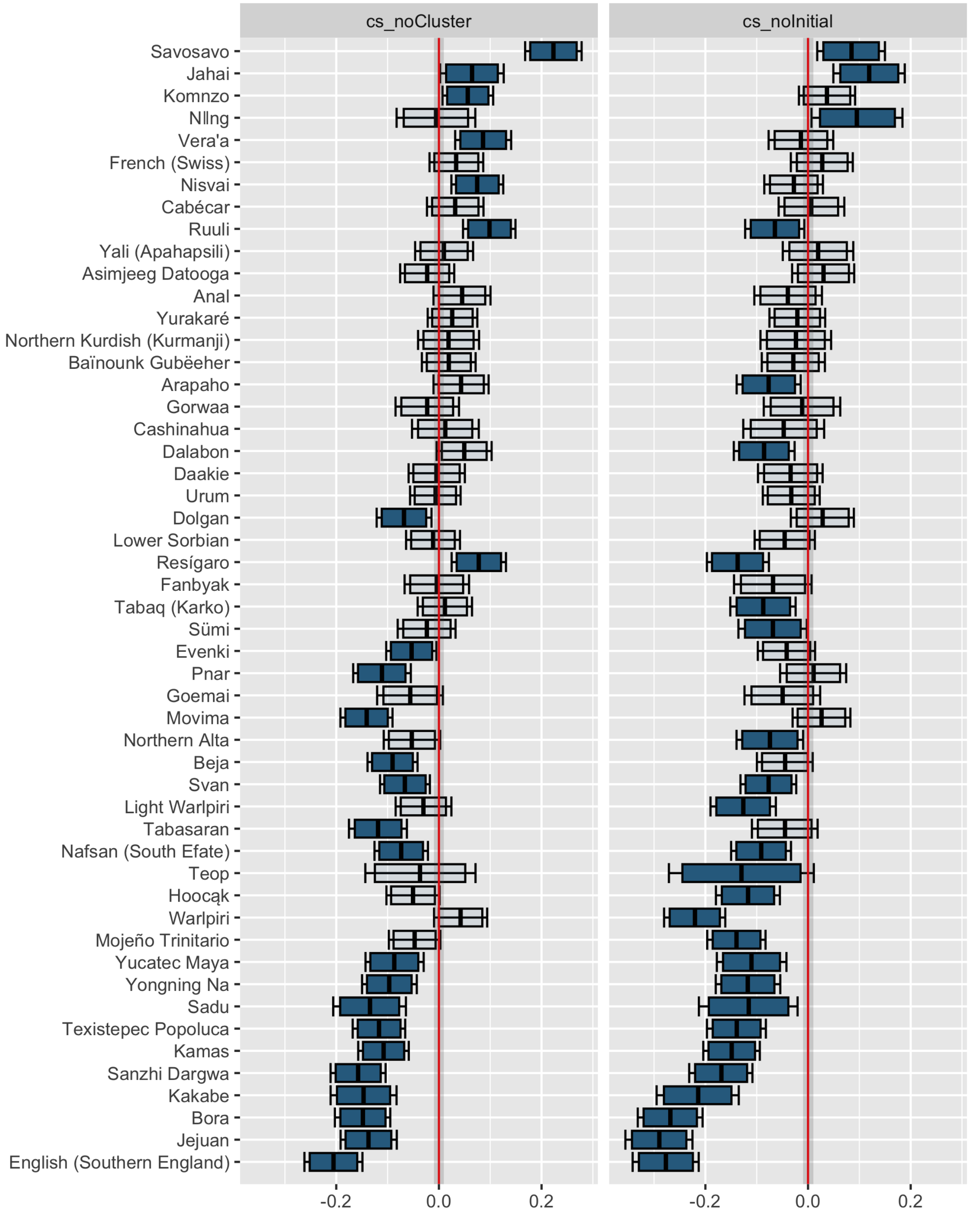

Figure 7: Posterior distribution for the control variable 'Consonant Cluster' across all languages of the sample. The intercept is modeled as consonants in the beginning of consonant clusters.

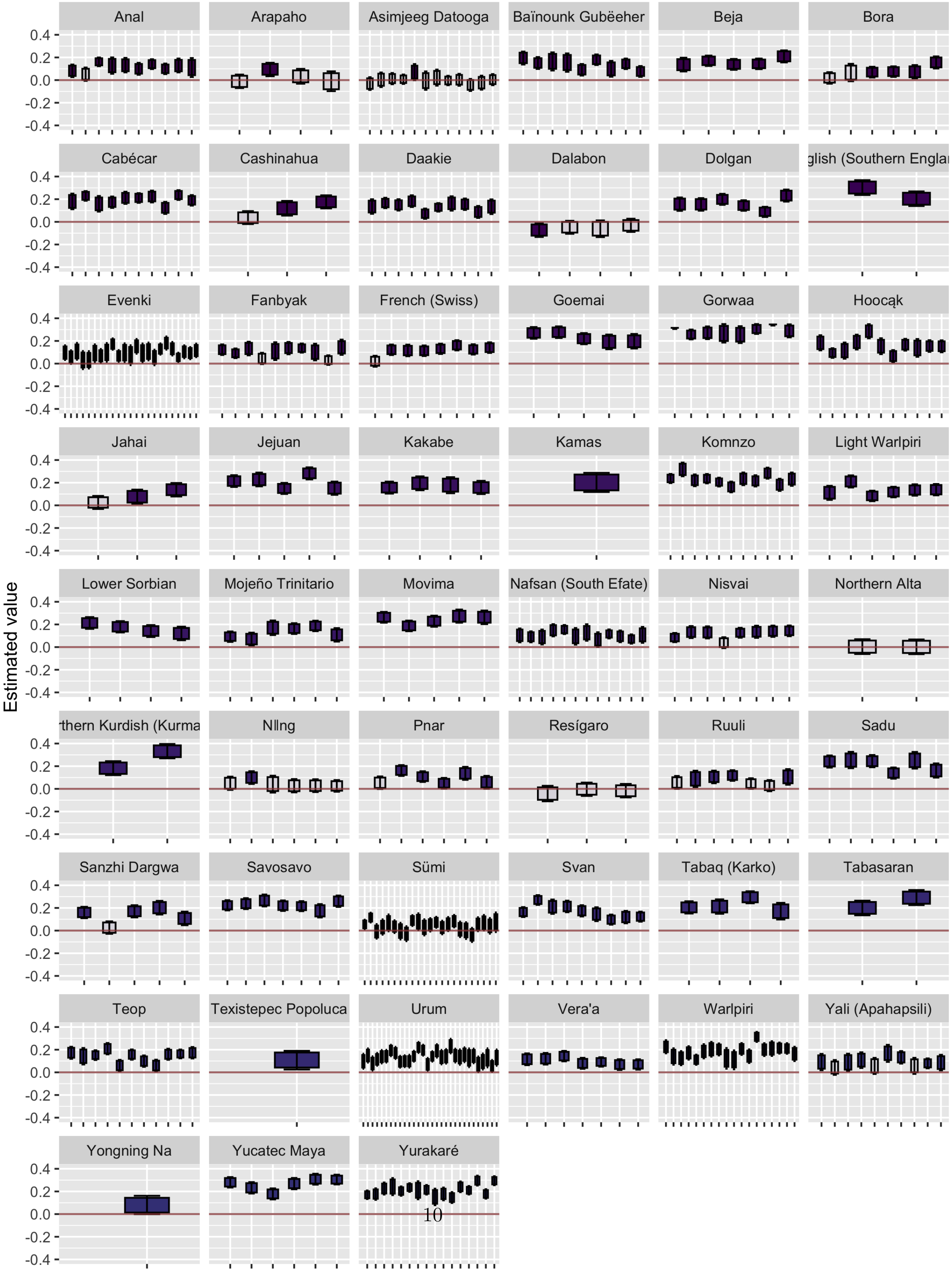

Figure 8: Word-initial lengthening for speakers of all languages.

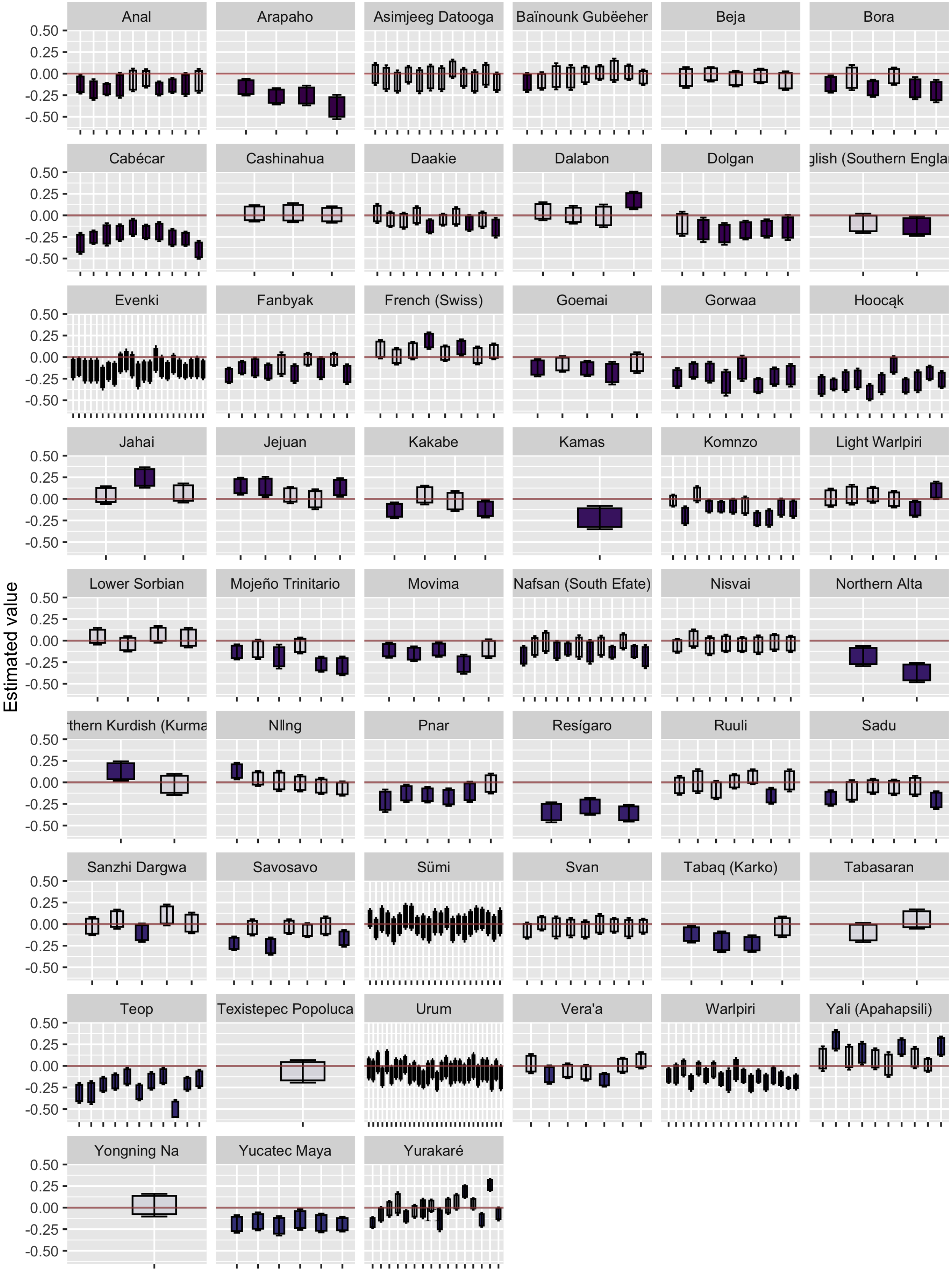

Figure 9: Utterance-initial lengthening for speakers of all languages.

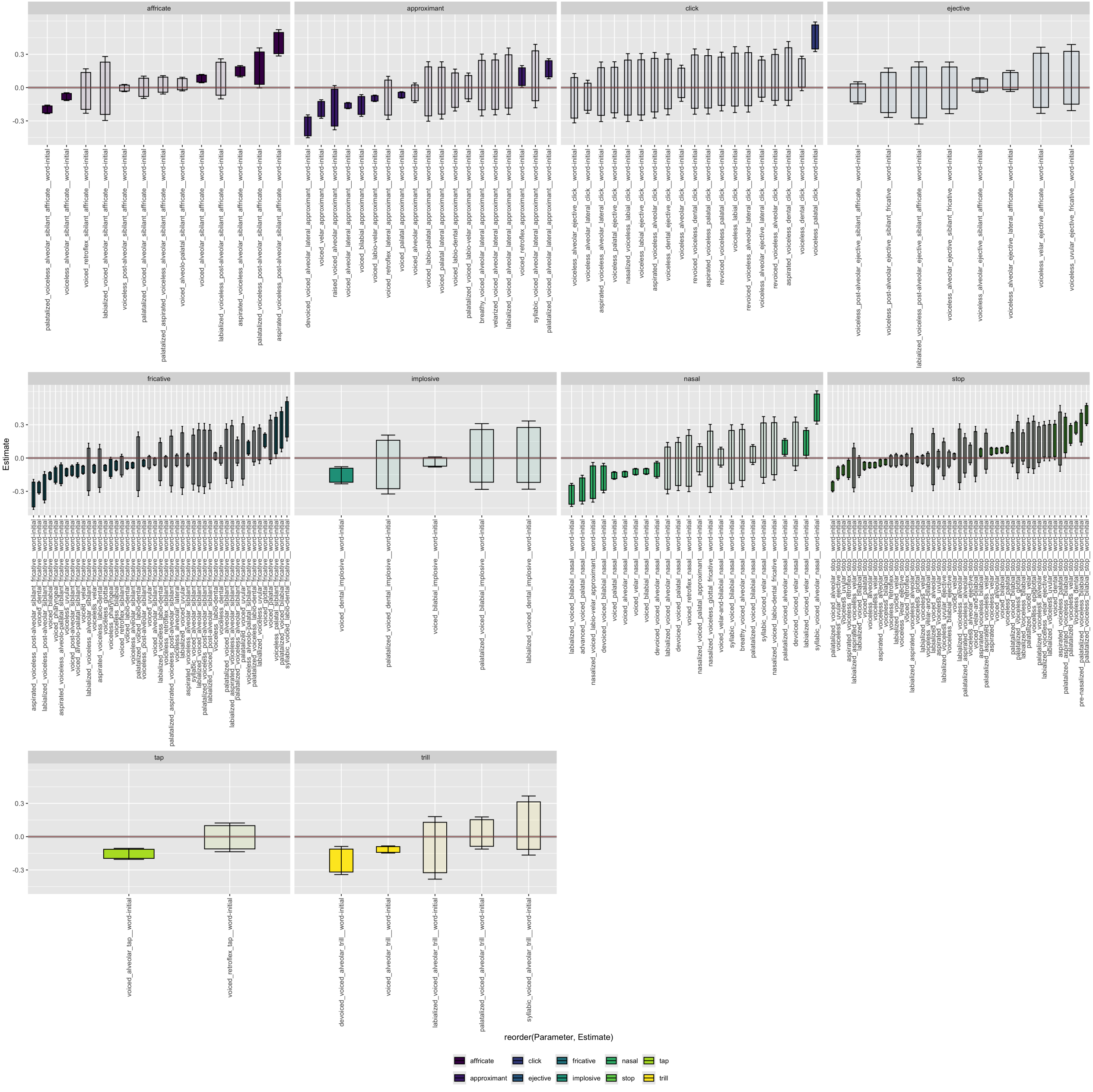

Figure 10: Lengthening of sound classes in word-initial position.

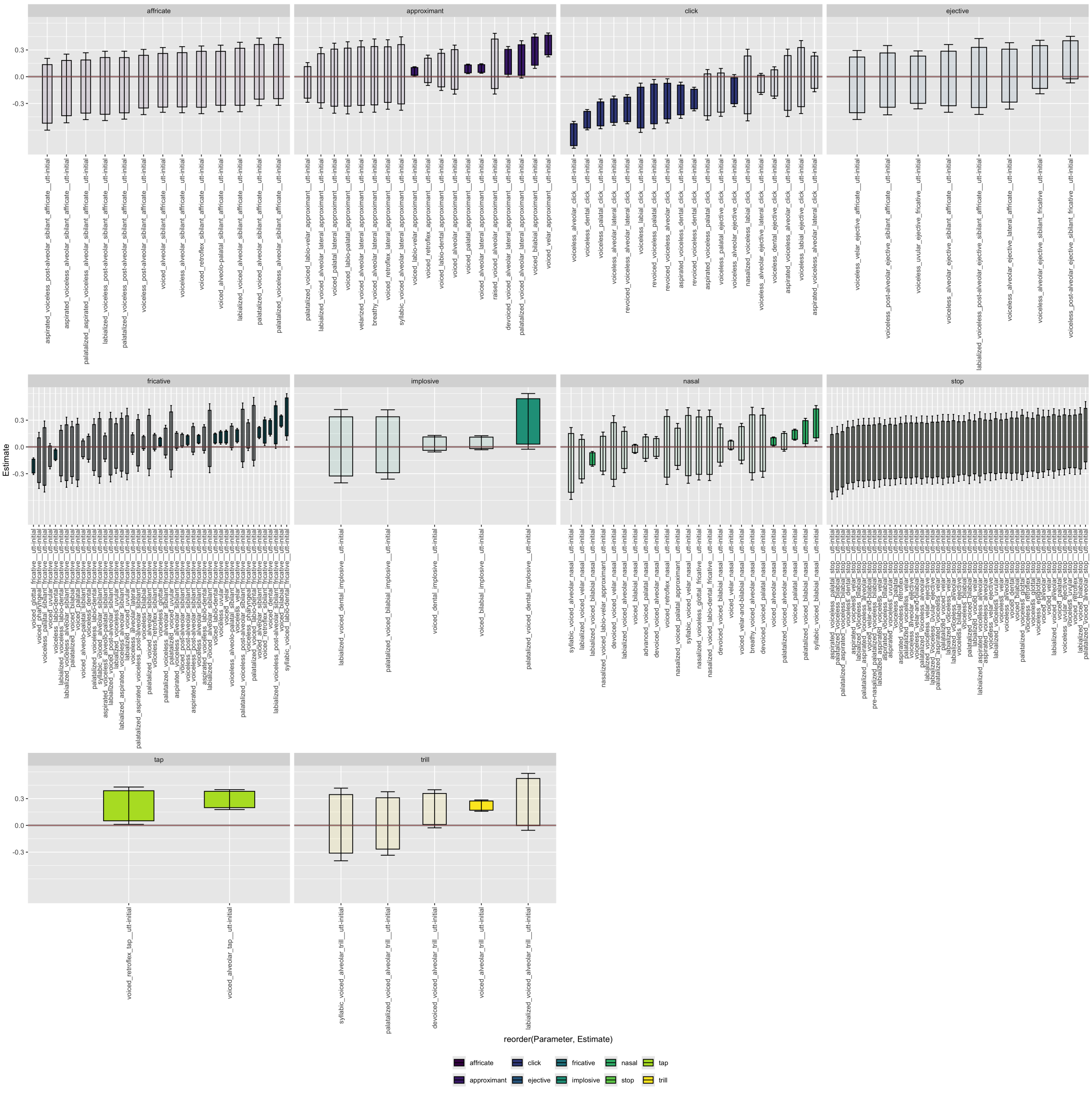

Figure 11: Lengthening of sound classes in utterance-initial position.

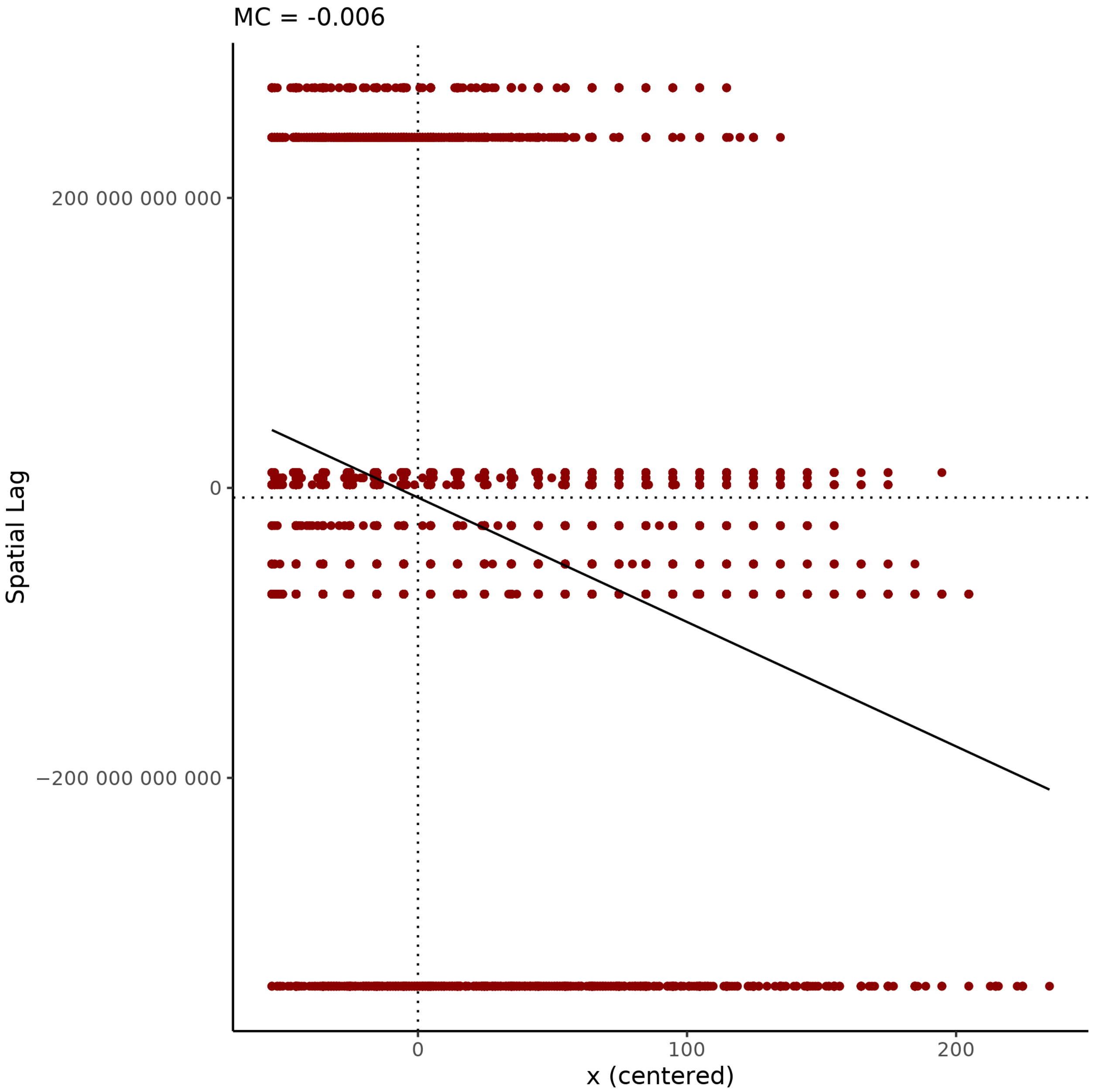

Figure 12: Moran coefficient for macro-area Africa.

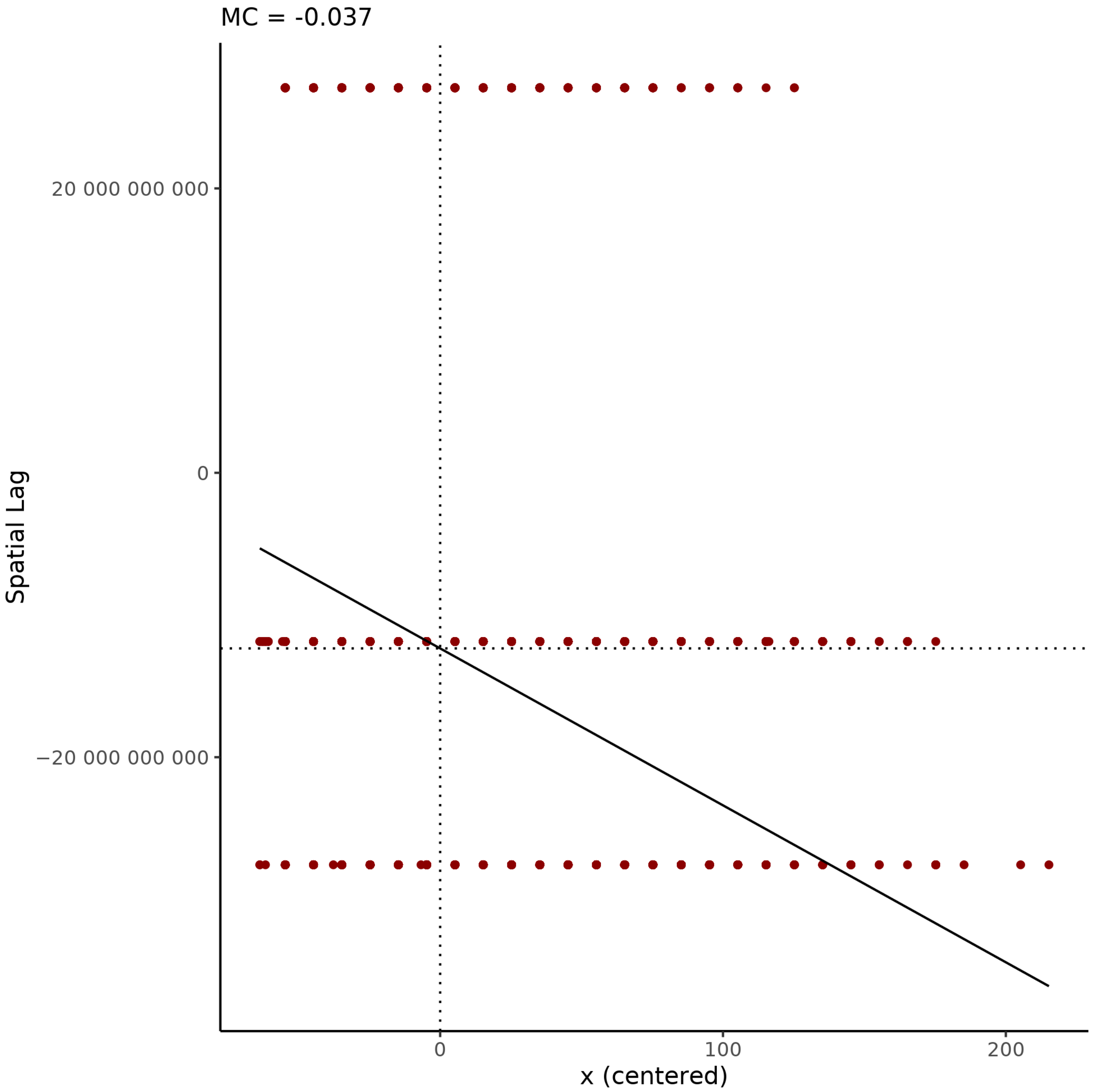

Figure 13: Moran coefficient for macro-area Australia.

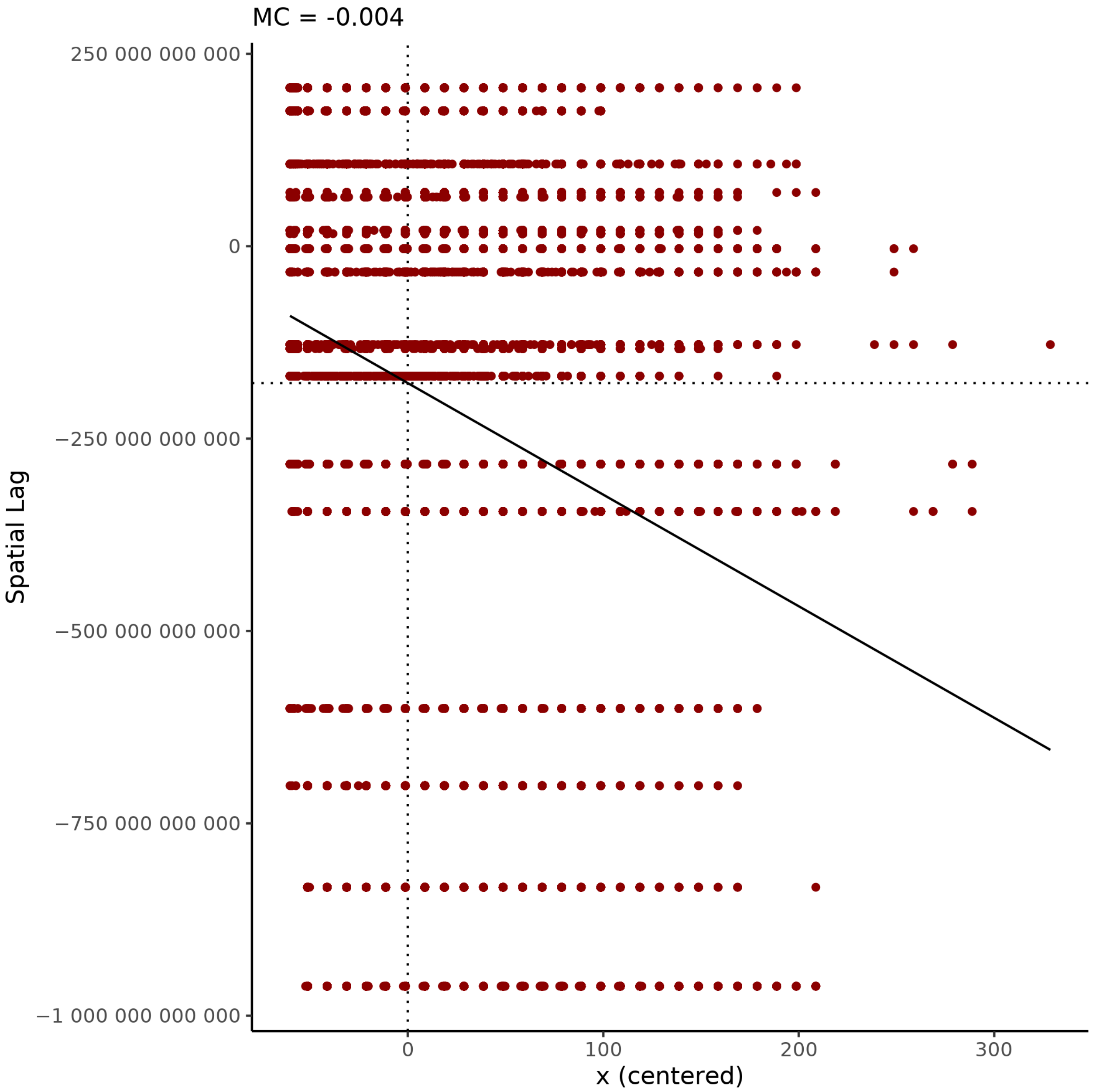

Figure 14: Moran coefficient for macro-area Eurasia.

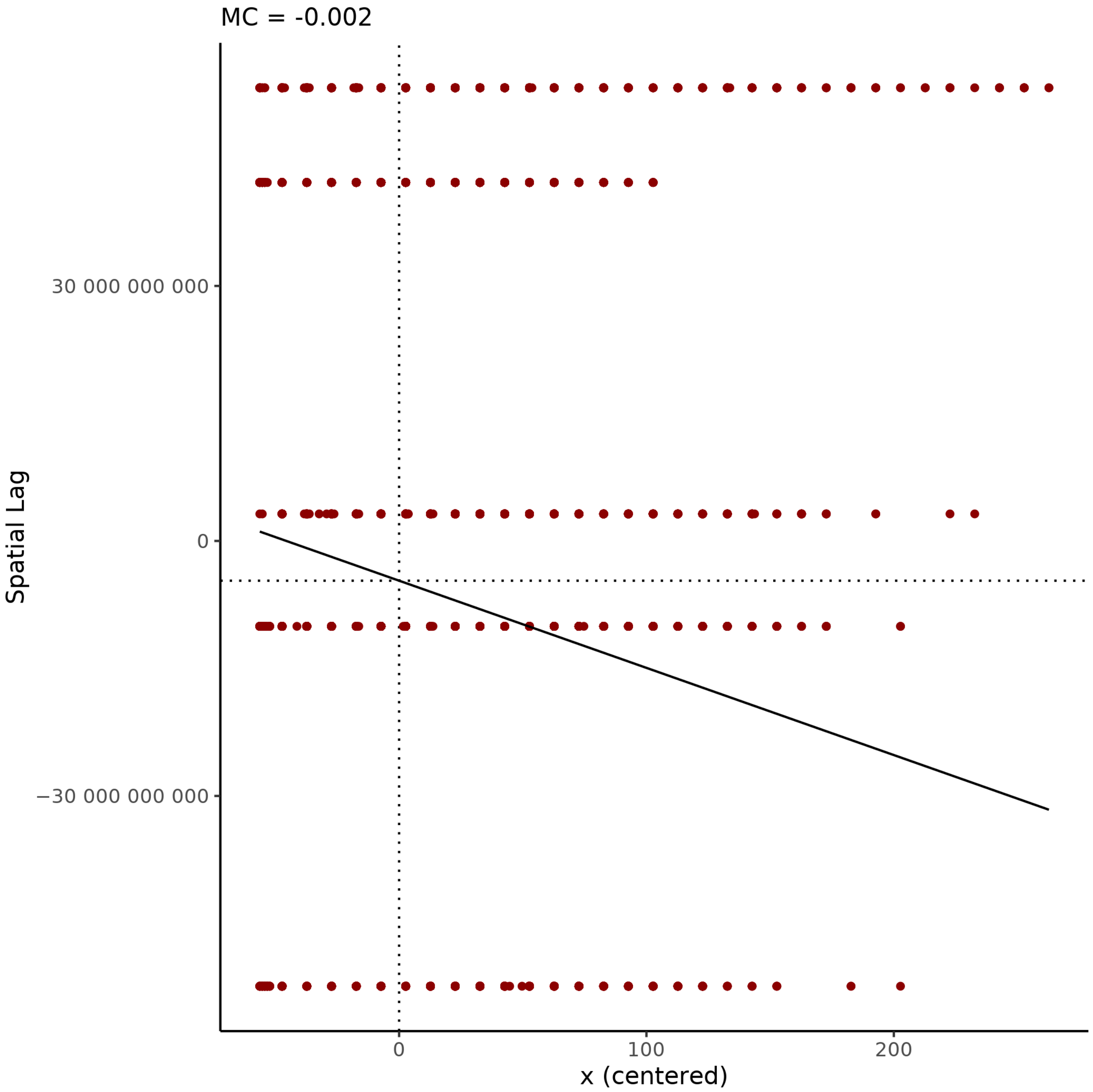

Figure 15: Moran coefficient for macro-area North America.

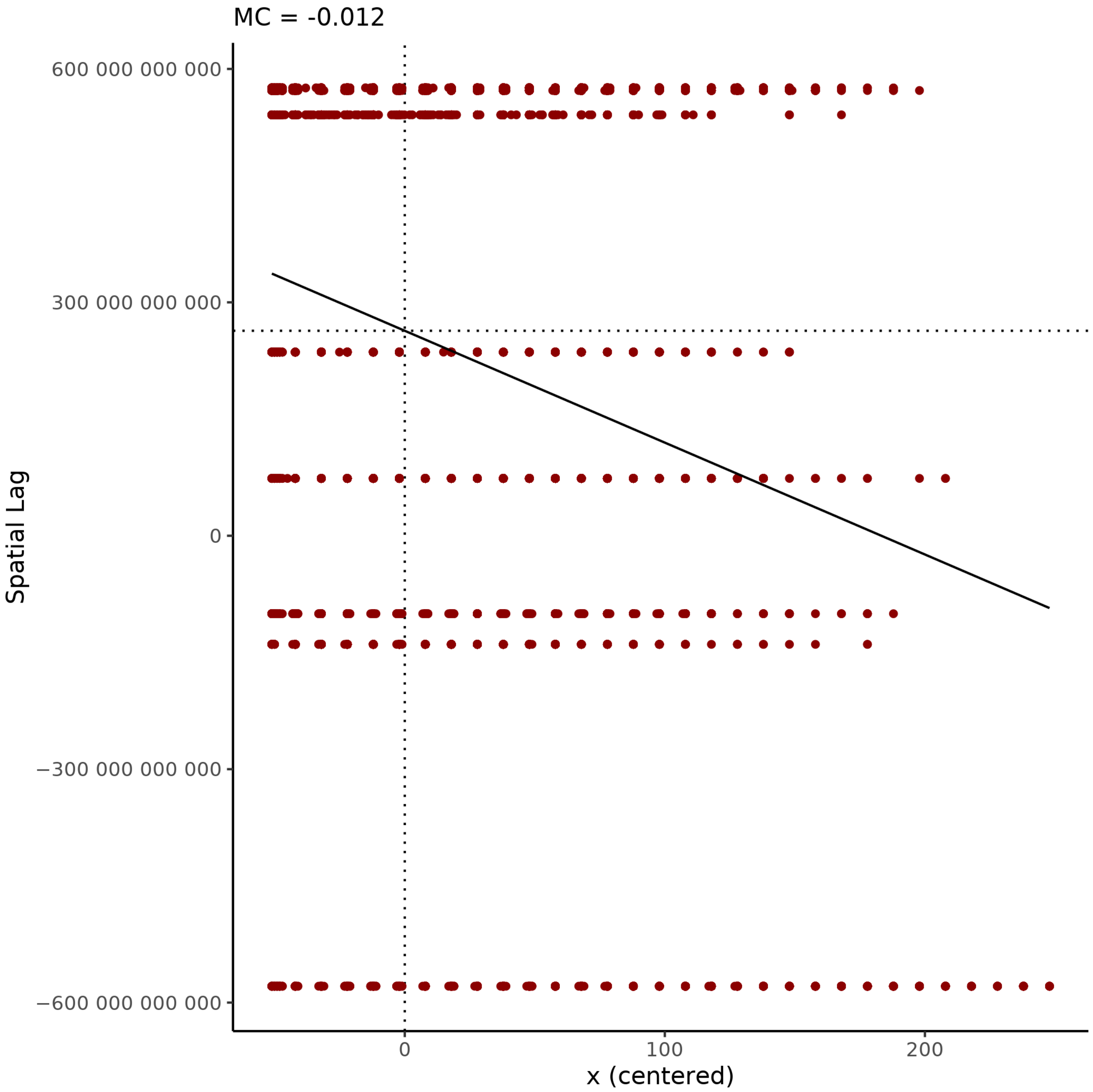

Figure 16: Moran coefficient for macro-area Papunesia.

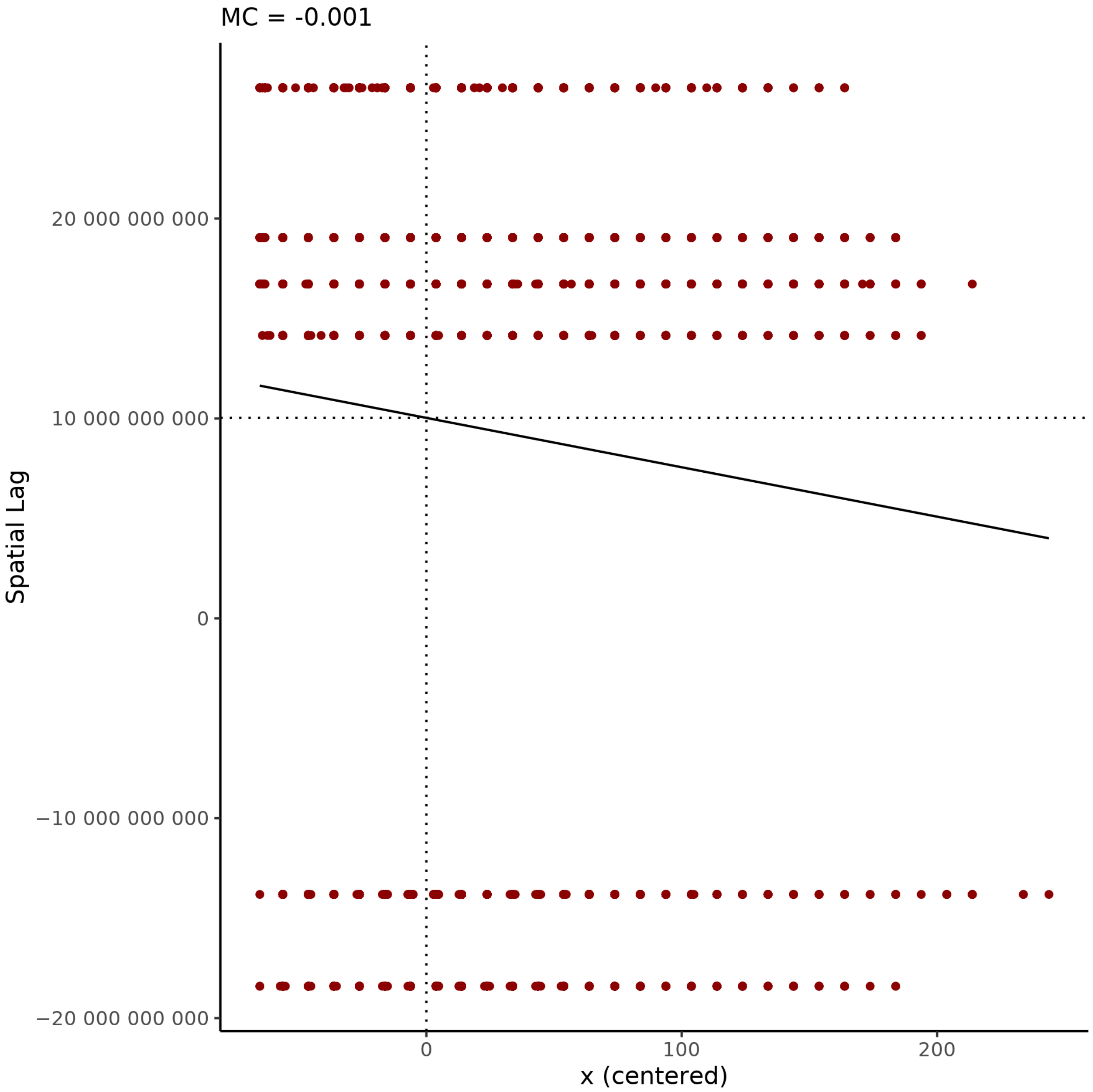

Figure 17: Moran coefficient for macro-area South America.

References

[1] Mikhail Alekseev and Sabrina Shikhalieva. *Tabasaranskyj jazyk*. Moscow: RAN, 2003.

[2] Trevor R. Allin. “A grammar of Resígaro”. PhD thesis. University of St Andrews, 1976.

[3] Niclas Burenhult. *A grammar of Jahai*. Vol. 566. Pacific Linguistics. Canberra: Research School of Pacific and Asian Studies, Australian National University, 2005.

[4] Chris Collins and Levi Namaseb. *A grammatical sketch of N|uuki with stories*. Vol. 25. Quellen zur Khoisan-Forschung/Research in Khoisan Studies. Cologne: Rüdiger Köppe, 2011.

[5] Andrew Cowell and Alonzo Moss Sr. *The Arapaho language*. Boulder, CO: University Press of Colorado, 2008.

[6] Anne Cutler. “Lexical stress in English pronunciation”. In: *The Handbook of English Pronunciation*. Ed. by M. Reed and J. M. Levis. Wiley, 2015, pp. 106–124. DOI: 10.1002/9781118346952.ch6.

[7] Christian Döhler. *A grammar of Komnzo*. Studies in Diversity Linguistics 22. Berlin: Language Science Press, 2018. DOI: 10.5281/zenodo.1477799.

[8] Caroline Féry. *Intonation and Prosodic Structure*. Cambridge, MA: Cambridge University Press, 2017.

[9] Diana Forker. *A grammar of Sanzhi Dargwa*. Berlin: Language Science Press, 2020.

[10] Jonah Gabry and Tristan Mahr. *bayesplot: Plotting for Bayesian Models*. R package version 1.10.0. 2022. URL: https://mc-stan.org/bayesplot/.

[11] Jonah Gabry, Daniel Simpson, Aki Vehtari, Michael Betancourt, and Andrew Gelman. “Visualization in Bayesian workflow”. In: *Journal of the Royal Statistical Society Series A: Statistics in Society* 182 (2 2019), pp. 389–402. DOI: 10.1111/rssa.12378.

[12] Alexandro-Xavier García-Laguía. “Documentation of Northern Alta: grammar, texts and glossary”. PhD thesis. Univesitat de Barelona, 2018. URL: http://hdl.handle.net/10803/664081.

[13] Richard Griscom. “Asimjeeg Datooga grammar sketch”. Manuscript, University of Oregon. 2018.

[14] Kenneth Hale and Josie White Eagle. “A preliminary metrical account of Winnebago acccent”. In: *International Journal of American Linguistics* 46.2 (1980), pp. 117–132.

[15] Andrew Harvey. “The Gorwaa noun: Toward a description of the Gorwaa language”. PhD thesis. University of London, 2018.

[16] Katharina Haude. “A grammar of Movima”. PhD thesis. Nijmegen: Radboud Universiteit Nijmegen, 2006.

[17] Birgit Hellwig. *A grammar of Goemai*. Vol. 51. Mouton Grammar Library. Berlin & Boston: De Gruyter Mouton, 2011.

[18] Birgit Hellwig and Gertrud Schneider-Blum. “Tabaq: In a State of Flux”. In: *Dotawo: A Journal of Nubian Studies* 1 (2014), Article 3.

[19] Thomas Kisler, Florian Schiel, and Han Sloetjes. “Signal processing via web services: the use case WebMAUS”. In: *Proceedings Digital Humanities*. Hamburg, 2012, pp. 30–34.

[20] Gerson Klumpp. “Kamas”. In: *The Oxford guide to the Uralic languages*. Ed. by Marianne Bakró-Nagy, Johanna Laakso, and Elena Skribnik. Oxford: Oxford University Press, 2022, pp. 817–843.

[21] Frank Kügler and Stavros Skopeteas. “Interaction of lexical tone and information structure in Yucatec Maya”. In: *Proc. 2nd International Symposium on Tonal Aspects of Languages (TAL 2006)*. 2006, pp. 77–82.

[22] Alexis Michaud. *Tone in Yongning Na. Lexical tones and morphotonology*. Berlin: Language Science Press, 2017. DOI: 10.5281/zenodo.439004.

[23] Saudah Namyalo, Alena Witzlack-Makarevich, Anatole Kiriggwajjo, Amos Atuhairwe, Zarina Molochieva, Ruth Gimbo Mukama, and Margaret Zellers. *A dictionary and grammatical sketch of Ruruuli-Lunyala*. African Language Grammars and Dictionaries 5. Berlin: Language Science Press, 2021.

[24] David George Nash. “Topics in Warlpiri grammar”. PhD thesis. Massachusetts Institute of Technology, 1980.

[25] Igor Nedjalkov. *Evenki*. London: Routledge, 1997.

[26] Pavel Ozerov. “Person indexation in Anal”. In: *Himalayan Linguistics* 18.1 (2019), pp. 26–53.

[27] Hiram Ring. “A grammar of Pnar”. PhD thesis. Nanyang Technological University, 2015.

[28] Françoise Rose. “Mojeño trinitario”. In: *Oriente*. Ed. by Pieter Muysken and Mily Crevels. Vol. 3. Lenguas de Bolivia. La Paz, 2014, pp. 59–97.

[29] Gunter Schaarschmidt. *A historical phonology of the Upper and Lower Sorbian languages*. Heidelberg: C. Winter, 1997.

[30] Stefan Schnell. “A grammar of Vera’a, an Oceanic language of North Vanuatu”. Manuscript, Universität Kiel. 2011.

[31] Amos Benjamin Teo. *A phonological and phonetic description of Sumi, a Tibeto-Burman language of Nagaland*. SEAsian Mainland Languages E-Series (SEAMLES). Canberra: Asia-Pacific Linguistics, The Australian National University, 2009.

[32] W. M. Thackston. *Kurmanji Kurdish: a reference grammar with selective readings*. Renas Media, 2006.

[33] Nicholas Thieberger. *A grammar of South Efate. An Oceanic language of Vanuatu*. Honolulu: University of Hawai’i Press, 2006.

[34] Wesley Thiesen and David Weber. *A grammar of Bora. With special attention to tone*. Dallas, TX: SIL International, 2012.

[35] Rik Van Gijn. “A grammar of Yurakaré”. PhD thesis. Radboud University Nijmegen, Faculty of Arts, 2006.

[36] Martine Vanhove. *Le Bedja*. Leuven & Paris: Peeters, 2017.

[37] Aki Vehtari, Andrew Gelman, Daniel Simpson, Bob Carpenter, and Paul-Christian Bürkner. “Rank-Normalization, Folding, and Localization: An Improved $\hat{R}$ for Assessing Convergence of MCMC (with Discussion)”. In: *Bayesian Analysis* 16.2 (June 2021). DOI: 10.1214/20-ba1221.

[38] Elisabeth Verhoeven. “Cabécar - a Chibchan language of Costa Rica”. In: *Amerindiana: Neue Perspektiven auf die indigenen Sprachen Amerikas*. Ed. by Jeanette Sakel and Thomas Stolz. Berlin: Akademie Verlag, 2012, pp. 151–169. DOI: 10.1524/9783050057682.151.

[39] Alexandra Vydrina. “A corpus-based description of Kakabe, a Western Mande language: prosody in grammar”. PhD thesis. Paris: INALCO, 2017.

[40] Claudia U. Wegener. *A grammar of Savosavo*. Vol. 61. Mouton Grammar Library 61. Berlin, Boston: Mouton de Gruyter, 2012. DOI: 10.1515/9783110289657.

[41] Søren Wichmann. "Underspecification in Texistepec Popoluca phonology". In: *International Journal of Linguistics* 27.2 (1994), pp. 455–473.

[42] Xianming Xu and Bibo Bai. "Sadu DoReCo dataset". In: *Language Documentation Reference Corpus (DoReCo) 1.2*. Ed. by Frank Seifart, Ludger Paschen, and Matthew Stave. Berlin & Lyon: Leibniz-Zentrum Allgemeine Sprachwissenschaft & laboratoire Dynamique Du Langage (UMR5596, CNRS & Université Lyon 2), 2022. DOI: 10.34847/nkl.3db4u59d. URL: https://doreco.huma-num.fr/languages/sadu1234.

[43] Chang-Yong Yang, Se-Jung Yang, and William O'Grady. *Jejueo: The language of Korea's Jeju island*. Honolulu: University of Hawaii Press, 2020.
