# Supplementary material for: Consonant lengthening marks the beginning of words across a diverse sample of languages
Source: Nat Hum Behav. 2024 Sep 24;8(11):2127–38. doi: 10.1038/s41562-024-01988-4 (PMC11576513; doi:10.1038/s41562-024-01988-4)
Supplement: Supplementary file 3 — Peer Review File [file 41562_2024_1988_MOESM3_ESM.pdf]

natureresearch

Peer Review Information

Journal: Nature Human Behaviour
Manuscript Title: Consonant lengthening marks the beginning of words across a diverse sample of languages
Corresponding author name(s): Frederic Blum

Reviewer Comments & Decisions:

Decision Letter, initial version:

6th March 2024

Dear Mr Blum,

Thank you once again for your manuscript, entitled "Consonant lengthening marks the beginning of words across a diverse sample of languages," and for your patience during the peer review process.

Your manuscript has now been evaluated by 3 reviewers, whose comments are included at the end of this letter. Although the reviewers find your work to be of interest, they also raise some important concerns. We are very interested in the possibility of publishing your study in Nature Human Behaviour, but would like to consider your response to these concerns in the form of a revised manuscript before we make a decision on publication.

To guide the scope of the revisions, the editors discuss the referee reports in detail within the team, including with the chief editor, with a view to (1) identifying key priorities that should be addressed in revision and (2) overruling referee requests that are deemed beyond the scope of the current study. We hope that you will find the prioritised set of referee points to be useful when revising your study. Please do not hesitate to get in touch if you would like to discuss these issues further.

1. Our reviewers raise some questions regarding the robustness of your results. To address these concerns, please conduct additional analyses and discuss potential dependencies between languages in your sample.

2. In addition, our reviewers make several suggestions for additional literature and theoretical models to be discussed. When revising your manuscript, please discuss these models as well as the theoretical

relevance of your findings. When discussing the theoretical relevance of your findings, we ask that you also make sure that unsupported claims are toned down or qualified as requested by Reviewer 3.

3. The reviewers raise some questions regarding the statistical models and their presentation. Please clarify the structure of the models and make revise the presentation of the results in response to the reviewer comments.

Finally, your revised manuscript must comply fully with our editorial policies and formatting requirements. Failure to do so will result in your manuscript being returned to you, which will delay its consideration. To assist you in this process, I have attached a checklist that lists all of our requirements. If you have any questions about any of our policies or formatting, please don't hesitate to contact me.

In sum, we invite you to revise your manuscript taking into account all reviewer and editor comments. We are committed to providing a fair and constructive peer-review process. Do not hesitate to contact us if there are specific requests from the reviewers that you believe are technically impossible or unlikely to yield a meaningful outcome.

We hope to receive your revised manuscript within two months. I would be grateful if you could contact us as soon as possible if you foresee difficulties with meeting this target resubmission date.

With your revision, please:

• Include a “Response to the editors and reviewers” document detailing, point-by-point, how you addressed each editor and referee comment. If no action was taken to address a point, you must provide a compelling argument. When formatting this document, please respond to each reviewer comment individually, including the full text of the reviewer comment verbatim followed by your response to the individual point. This response will be used by the editors to evaluate your revision and sent back to the reviewers along with the revised manuscript.

• Highlight all changes made to your manuscript or provide us with a version that tracks changes.

Please use the link below to submit your revised manuscript and related files:

[REDACTED]

Note: This URL links to your confidential home page and associated information about manuscripts you may have submitted, or that you are reviewing for us. If you wish to forward this email to co-authors, please delete the link to your homepage.

We look forward to seeing the revised manuscript and thank you for the opportunity to review your work. Please do not hesitate to contact me if you have any questions or would like to discuss these revisions further.

Sincerely,

[REDACTED]

Reviewer expertise:

Reviewer #1: initial consonant lengthening / word-initial lengthening

Reviewer #2: initial consonant lengthening / word-initial lengthening

Reviewer #3: Bayesian models ; linguistics

REVIEWER COMMENTS:

Reviewer #1:
Remarks to the Author:
Review on Blum et al.
"Consonant lengthening marks the beginning of words across a diverse sample of languages"
by Gerrit Kentner

The present manuscript provides an analysis of word-initial consonant lengthening comparing utterance-initial and utterance-medial word positions in a relatively large and diverse sample of understudied languages (n=51). The results afford convincing evidence that the word-initial position is phonetically privileged to be the target of lengthening in the vast majority of, if not in all, languages. At the same time, the study shows that utterance-initial words are exempt from, or at least not systematically affected by, the potentially universal word-initial lengthening effect.

The manuscript is well-written, easy to follow, succinct, and clear. The methodological approach is sound and meets the high standards required for such a study (and for the target journal). The code and the data are provided as supplementary material so that the analysis is reproducible.

The particular merit of this study lies in its methodological rigor and empirical breadth, which goes far beyond similar studies on a few well-researched languages. Harnessing a corpus of lesser-documented languages makes it possible to persuasively suggest a universal phonetic effect of word-initial lengthening. The functional significance of this effect, namely for segmenting the continuous speech stream into a string of discrete meaningful units, is discussed in the context of extant literature on the topic. The manuscript thus provides an important key to our understanding of the formidable and fundamental task of finding linguistic structure in the acoustic tangle of speech. The paper is therefore of interest to linguists, speech scientists, psychologists and, given the possible universality of the phenomenon, anthropologists in general.

I have only a small number of relatively minor comments that the authors could take up in an updated version:

General question regarding the data: Some of the languages in this sample might have (lexical) stress, or even systematic word-initial stress. Is there a way to disentangle effects of stress on word-initial phones from pure word-initiality?

Line 074 et seqq
"Languages differ in their inventory and combinations of such acoustic
cues [10], but one particularly widespread correlate for the prosodic organization of
speech into different domains are the acoustic properties of word-initial phones [1, 11]."
The references to Cutler and Krivokapić do not really support the claim that the phenomenon under investigation (acoustic properties of word-initial phones) is “particularly widespread”, since their investigations are mostly confined to English.

Line 095 et seqq
"However, the reality and magnitude of
initial lengthening is still debated, and previous findings could not always be reliably
reproduced [17]."
The reference to Wightman et al. (1992) seems inaccurate, as this study wasn’t concerned with initial lengthening but final lengthening.

Line 101 et seq.
"In previous studies, utterance-initial consonants
have been found to be lengthened or shortened, but with an overall small change
in duration [18–20]."
Reading Amir et al. (2004), it appears that they look at initial syllables rather than consonants. Cruttenden (1997) discusses the phenomenon of “anacrusis”, i.e. shortening of unstressed utterance-initial syllables. Since the authors refer specifically to the segmental level (consonants), these references

might not be fully appropriate.

Paragraph beginning in line 126
The purpose of this paragraph for the story presented here is not clear to me. The cohort model is not so much about word segmentation, rather about word identification, no? The asymmetry between word onset consonants and other consonants is more important for word segmentation than for word identification, I think.
"Consequently, wordinitial
positions are assigned a prominent status in phonological theories [25–28]."
The word “consequently” is inappropriate here. Surely, the prominent status of word-initial consonants within phonological theories is not based on the cohort model, nor on speech processing more generally.

Statistical model – Figure 2, page 6
The factors “utterance-initial” and “word-initial position” are represented as “varying effects” (grey arrows). Looking at the model in the github repository, these factors were specified as fixed effects for which there are varying slopes in the random effects structure of the model. The other “varying effects” (grey arrows) were entered as random effects. I am not sure about the terminology applied here, but there seems to be a difference between “random effects” and “varying effects”, or at least it seems that the grey arrows in Figure 2 conflate or confound two different types of effect in the model. Please clarify.
I was curious to see that “sound class” was entered as random effect (explanation Line 720-727). The authors assume six different sound classes ((un)voiced plosive, (un)voiced fricative, (un)voiced sonorant). The difference between continuants (fricatives, sonorants) and non-continuants (plosives) could be construed as a fixed effect, since there is a relatively clear hypothesis that non-continuants are shorter than continuants. So at least this dimension would not properly be construed as “random” (I have no clear intuition/hypothesis regarding the durational distinction between fricatives and sonorants, or between voiced and unvoiced sounds).

Line 496 et seqq.
"… that lengthening is more closely linked to the identification
of word units than to prosodically structuring speech into larger units such
as prosodic phrases."
Here, the authors might want to specifically refer to “initial lengthening”, as it is known that final lengthening is definitely linked to prosodic structuring.

Line 539 et seqq.
"As lengthening
can be one phonetic manifestation of phonological strength, this might suggest that

we should find consistent differences in lengthening across sound classes."
The concept of “phonological strength” and its relation to length is not really clear to me. I could imagine that weakening might likewise lead to an increase in duration (think of lenition processes, such as spirantisation of underlying plosives).
A stylistic remark: the word “consistent” appears three times within this paragraph (four times on this page).

Line 555 “be” --> “to”
final segments have been shown be undergo considerable lengthening

Line 553-563
Discussion about the differently expressed demarcative functions of initial and final lengthening.
There is another interesting parallel with final lengthening: Recent evidence suggests that, at very strong prosodic boundaries with long pauses, the final lengthening-effect vanishes (see Kentner et al. 2023), just as the word-initial lengthening effect is absent when the word commences an utterance. In both cases, this could be explained by an avoidance of redundant marking of boundaries (or lack of functional ambiguity due to a clear pause). [this study is part of my own work, please do not feel obliged to cite]

Discussion section
The results suggest that word-initial lengthening is just word-initial and not related to higher levels of prosodic structure. This finding thus affords good reasons to discuss a prominent model in articulatory phonology, namely the pi-gesture model. According to Byrd and Krivokapić (2021), this model assumes that final lengthening and initial lengthening are different phenomena that are due to a single process, namely the pi gesture, which somehow acts as a pacemaker, slowing down articulatory gestures around prosodic breaks; the stronger the break, the greater the slowdown.
Byrd and Krivokapić (2021: 37)
"Within the π-gesture model, boundaries are represented gradiently rather than categorically or symbolically. So, boundaries encoding greater disjuncture (for example, in traditional terms, at an Intonational Phrase versus an Intermediate Phrase) have greater activation and thereby exert a greater slowing or “stretching” of the concurrent gestural activations. The π-gesture model makes no distinction between final and initial phrase edges; a single π-gesture spans a boundary, reaching transgesturally into both final and initial phrase-edge material."

The authors of the present paper might want to respond to these considerations, as their results seem to be relevant in this context.

Line 670 (typo “used / using”)

References
Byrd, D., & Krivokapić, J. (2021). Cracking prosody in articulatory phonology. Annual review of linguistics, 7, 31-53.

Kentner, G., Franz, I., Knoop, C. A., & Menninghaus, W. (2023). The final lengthening of pre-boundary syllables turns into final shortening as boundary strength levels increase. Journal of Phonetics, 97, 101225.

Reviewer #2:
Remarks to the Author:
General comments

This is a worthwhile and original analysis of a wide range of languages which examines an important source of durational variation, one that is likely to be a segmentation cue for speakers across languages, and may indeed be universal (e.g., White, Benavides-Varela & Mády, 2020).

The data-sets are not well matched between languages, with samples ranging from 1 to 30 speakers and from less than 2000 words to more than 9000. This is not intrinsically a problem, as the key point here is not a comparison between languages, although cross-linguistic comparisons are reported (Figures 3 and 4). There are, thus, various limitations on the interpretations of observed cross-linguistic differences and the authors are rightly circumscribed about this, with a number of confounding factors noted in the discussion.

Sources of durational variation between languages may also relate to distributional factors. As discussed below, the authors need to more clearly account for: firstly, singleton consonants vs those in initial clusters (if these were distinguished, it was not clear from the methods); secondly, intrinsic durational variation due to place of articulation, which is not controlled here (see 720 point below).

It is a strength of the study that most languages analysed here are previously understudied as regards their durational properties. The inclusion of varieties of French and English does allow some checking of methods and analyses, however. There are many empirical studies of English speech timing, for example, and the magnitude of effects reported in the current paper for English (both word-initial and utterance-initial) could instructively be compared with those of earlier studies (for original sources, see reviews in, e.g., Fletcher, 2010; White, 2014).

Overall, this paper deserves to be widely read, given the implications for the understanding of phonetic universals. Such findings inform a wide range of theoretical domains, including language evolution and

individual language development, and have obvious implications for language technologies.

Specific points are indexed by line numbers in the manuscript.

Introduction

para beginning 113: The discussion of potential phonotactic cues to word segmentation)"[language-] specific complex clusters") is rather convoluted and could be shortened: some languages have effective consonant clustering cues and some do not.

Methods

General: How were consonant clusters treated? These should definitely be analysed separately from singletons in onset position. Effects of coarticulation as well as variation in the distribution of prosodic lengthening effects (see 705 point below) mean that intrinsic durations in cluster onsets will tend to differ from those in singleton onsets. Such considerations (see also point 720 below) may well help to explain the observations of two languages that apparently lack word-initial lengthening in these data.

682: Please could you give an indication of lower outlier thresholds? Were these, in practice, at 0ms or were they above that?

705: Note that "polysyllabic shortening" has been shown, at least for English, to be modulated by higher-level prosodic lengthening effects, i.e., the inverse relation between word length and the duration of sub-units (such as the primary stressed syllable) only holds when the word is subject to phrasal accent or final lengthening (e.g., White & Turk, 2010; White, 2014; also, e.g., Suomi, 2009, for Northern Finnish).

720: "we also control for variation of the effect across different sound classes, since there might be variation in the elasticity of segments depending on whether they are stops (and affricates), fricatives, or sonorants." There should also be control for place of articulation, as durational differences between bilabial, alveolar and velar stops (for example) are systematic and well-attested (e.g., Klatt, 1976). Moreover, differences are not simply due to variation in "elasticity", but also due to intrinsic articulatory constraints. As distribution of consonant types is not controlled between location (word-initial vs word-medial), and is moreover unlikely to be balanced (due to language-specific positional phonotactics), phoneme-level distinctions should be modelled at the most finely-detailed level permitted by the data.

Results

221: “Varying slopes and intercepts have been included in a nested way for speakers and different sound classes for each language.” Please clarifying. What is nested within what else?

Discussion

General: The listener requirement for cues to both word segmentation and word recognition/lexical access, both of which are boosted by word-initial lengthening, is very likely to contribute to the apparent universality of word-initial lengthening (see White, Benavides-Varela & Mády, 2020, for extended discussion). That specific linkage - i.e., between segmentation and recognition - could be discussed at more depth here.

486-499 (also 108): The functional motivation for the lack of consistent utterance-initial lengthening was formulated earlier than the reference cited ([15], at line 108): thus, see White, 2002; White 2014.

Typos
670: “using WebMAUS service was used”

Additional references

Klatt, D. H. (1976). Linguistic uses of segmental duration in English: Acoustic and perceptual evidence. The Journal of the Acoustical Society of America, 59(5), 1208-1221.

Suomi, K. (2009). Durational elasticity for accentual purposes in Northern Finnish. Journal of Phonetics, 37, 397-416.

White, L. (2002). English speech timing: A domain and locus approach. University of Edinburgh PhD dissertation.

White, L., & Turk, A.E. (2010). English words on the Procrustean bed: Polysyllabic shortening reconsidered. Journal of Phonetics, 38, 459-471.

Reviewer #3:
Remarks to the Author:
Summary: This article investigates word-initial lengthening, i.e. referring to the phenomenon when consonants at the beginning of a word are longer than in word medial and final position. The authors investigate this phenomenon in a sample of (semi)spontaneous speech across 51 languages. They report robust evidence for word-initial lengthening within their sample.

A unique strength of this paper is that it investigates a diverse (and for standards in the field large) sample of mostly non-WEIRD languages. Analyses such as the present one have the potential to provide valuable estimations of effect magnitude and variance across language (for the phenomenon under scrutiny). The authors also make their scripts and data processing steps open and transparent for the reader to evaluate. I tip my head to the transparency demonstrated here.

Having said that, the paper has room for improvement: I suggest (1) toning down overconfident claims (or at least qualifying them), (2) spelling out the theoretical relevance of their findings, (3) discussing and taking into account possible dependencies between languages in their sample, and (4) presenting the evidence in a more appropriately conservative way.

Detailed comments below:

1. Overclaiming
The paper overconfidently presents evidence at times and I'd invite the authors to nuance their statements (or directly qualify them more often).

E.g. "one particularly widespread correlate for the prosodic organization of speech into different domains are the acoustic properties of word-initial phones" (ll. 76f)

Not only is the cross-linguistic coverage of this phenomenon narrow at best (and that is exactly why the present paper is a great contribution), but the evidence itself is also often based on experiments with only a few speakers. Please either nuance or qualify your statement.

E.g. "Despite these limitations, the robust patterns across a word-wide sample of languages suggest a statistically universal process of lengthening of word-initial consonants." (ll. 631f)
See below. I suggest to nuance this very strong claim the universality of the phenomenon in light of what we can actually learn from the data. I do not believe the paper needs to claim universality to be a valuable contribution.

2. Theoretical relevance
The paper leaves the assumed impact of the phenomenon vague, so I would like to invite the authors to make their claims more explicit.

E.g. "A compelling rationale for word-initial lengthening effects is the cohort model that describes the incremental processing of speech" (ll. 126f.). First, the cohort model does not describe but propose incremental processing mechanisms and it does so specifically for spoken word recognition. Second, the

phrasing of this sentence leaves much room for interpretation. Do the authors propose that because humans seem to incrementally recognize words (i.e. as acoustic information comes in, lexical competitors compatible with the acoustic information are activated and incompatible ones are eliminated), acoustically marking word boundaries by durational cues helps that process? If so how exactly does initial lengthening affect this process? If there are other assumptions that the authors make, please specify the causal relationships you assume/propose. The authors continue two sentences later with “and asymmetries in the distributions of sounds play an intricate part in marking domain boundaries“ (ll 133f). I do not fully understand this sentence: Asymmetries in the distribution of durational values? I recommend clarifying.

On lines 462f. the authors state “Our findings carry significant implications for understanding the incremental nature of lexical processing in natural human speech.” I am puzzled by this statement. This paper does not show any link between initial lengthening and lexical processing. It is an assumption made by the authors (which also remains vague). The authors continue: ”Longer consonantal segments at the onset of words may thus constitute one of the most fundamental strategies for the segmentation and identification of distinct linguistic units”. This claim, again being very detached from the actual data presented, suggests that the authors actually mean that initial lengthening helps segmenting utterances into smaller units such as words. Albeit related, this is not the same as incremental processing in spoken word recognition as suggested in the introduction. I think the paper would benefit from nuancing these statements more and making explicit what causal mechanisms are assumed here exactly, as well as how the data do or do not speak to these claims.

While the assumption that prosodic cues help listeners to segment speech is not controversial, certain (rather small) acoustic asymmetries such as initial lengthening might also be mechanical consequences of how articulatory gestures are organised and coordinated. Whether the acoustic consequences of such assumed asymmetries are perceptually exploited across these languages remains an open question.

3. Sampling
I would like to see more information and consideration of the sample. We know that languages are not independent data points due to areal and genetic relationships. This has been a common methodological bottle neck for using cross-linguistic data in inference. E.g three related languages showing the same property tell us little about the generalisability of that property and should inferentially be taken into account (e.g. Roberts et al. 2015). I would like the paper to present (a) more information about possible dependencies between languages and (b) at least discuss the possible impact of these dependencies on their inferential procedure. Does the “robustness” of the pattern hold up when controlling for areal and genetic dependencies between languages? (but see my concerns about robustness below).

Roberts, S. G., Winters, J., & Chen, K. (2015). Future tense and economic decisions: Controlling for

cultural evolution. PloS one, 10(7), e0132145.

4. Interpretation of evidence
The authors interpret the results of their Bayesian model as evidence for a robust pattern across languages.

I have several caveats with regard to this interpretation and the presentation of it. Note that I do believe the authors that we see a level of consistency that is worthwhile discussion, but I think it is worth being more humble when interpreting the results (which does not make the observation weaker at all, only more honest). I do not doubt that the data seem descriptively compelling and given the immense variability observed in spontaneous speech, that is sometimes as much as we can hope for, but the inferential presentation is in my opinion overconfidently presenting the results:

The inference solely relies on the interpretation of the population estimates and the estimated uncertainty around it. The credible intervals are interpreted relative to two reference values: point zero and the boundaries of a ROPE (which is not exactly defined in the paper as far as I can see, see below).

First, this form of inference is very dependent on the specified model, so generally not a very strong measure of evidence but it is not uncommon that researchers use this form of inference.

Second, the authors relate the reference values to the 80% HDI interval and claim that most languages' 80% HDIs are outside of said reference values. This is very conservative. Concretely it means for more than a few languages, a substantial amount of plausible predicted values cross the reference values. I recommend using a more conservative comparison with e.g. the 95% HDIs and nuance the authors' confidence.

Third, I would like to see the ROPE clearly defined and theoretically justified. I was puzzled that on the one hand the authors mention an important perceptual threshold, the just noticeable difference which lends itself very well to be used as a ROPE, but apparently did not use that threshold as the ROPE (even though they do argue for the perceptual relevance of their effect magnitude).

Finally, the central figure 3 might be again overconfidently representing the nature of the data. The graph shows model estimates pulled from a hierarchical model, i.e. shrinkage will pull individual estimates towards the grand mean and the posterior distribution are much narrower than the empirical ones. I think readers would benefit more from a graph that plots (either additionally or separately) the raw data distributions in order to see if the data also suggest a unidirectional pattern.

In light of the omnipresent but healthy uncertainty in speech data, I suggest the authors revisit some statement in their discussion that are interpreting null results. Null results, particularly within an analysis

using their inferential process, are not informative whatsoever, so I recommend rephrasing these statements.
E.g. ll 544f. “The effect is consistent across all sound classes, despite their differences in mode and articulation. The effect is also consistent between all speakers of our data, which points to a uniform effect beyond individual speakers and languages.”
Both of these statements interpret the absence of “robust” effect estimates underlapping with 0 (or their ROPE) as evidence of consistency. They are merely compatible with this interpretation but also not incompatible with alternative point estimates that are non-zero.

5. Misc

”Clusters cannot be used as a universal method for the purpose of segmenting the beginning of a word…” (ll. 121f.) Do you mean clusters cannot be used as a domain of measurement?
Reference 24 is not the appropriate reference for its preceding sentence.
Neither figure 1a nor figure 1c have readable text elements. I recommend making those figures more accessible by increasing the font size

I recommend using a better contrast for the 80% HDI boxes in figure 3. The black median is difficult to see on the dark purple background.

ll 522f. The term s-mobile is likely unfamiliar to many potential readers.

ll 557f. “However, evidence for final lengthening at the word level remains dubious at best”. Please qualify this statement. Some people might say that evidence for initial strengthening and lengthening is also dubious at best given the small and unjustified sample sizes in these experiments.

The authors note that they use word frequency as a covariate (ll 695f.). Where did they take these frequencies from?

The authors note that their covariates are highly correlated. Correlated predictors lead to collinearity which can be a problem when interpreting coefficients. (e.g. Tomaschek et al. 2018). How did the authors tackle this potential bias?

ll 676f.: “We also removed non-pulmonic consonants such as clicks, implosives and ejectives due to the large differences in articulation, compared to pulmonic consonants.“ I must have missed something, because I do not understand the justification for removing these consonants. Them being different should not affect the relative lengthening of them in certain phonotactic positions.

ll 682f. ”Lastly, we excluded outliers beyond three standard deviations of the mean for each speaker.“ This decision artificially reduces variance. Can the authors justify this choices? Even in a perfect normal distribution, 0.3% of data fall outside of 3 SDs.

Refs:

Tomaschek, F., Hendrix, P., & Baayen, R. H. (2018). Strategies for addressing collinearity in multivariate linguistic data. Journal of Phonetics, 71, 249-267.

Author Rebuttal to Initial comments

REVIEWER COMMENTS:

Reviewer #1:

Remarks to the Author:

Review on Blum et al. "Consonant lengthening marks the beginning of words across a diverse sample of languages"

The present manuscript provides an analysis of word-initial consonant lengthening comparing utterance-initial and utterance-medial word positions in a relatively large and diverse sample of understudied languages (n=51). The results afford convincing evidence that the word-initial position is phonetically privileged to be the target of lengthening in the vast majority of, if not in all, languages. At the same time, the study shows that utterance-initial words are exempt from, or at least not systematically affected by, the potentially universal word-initial lengthening effect.

The manuscript is well-written, easy to follow, succinct, and clear. The methodological approach is sound and meets the high standards required for such a study (and for the target journal). The code and the data are provided as supplementary material so that the analysis is reproducible.

The particular merit of this study lies in its methodological rigor and empirical breadth, which goes far beyond similar studies on a few well-researched languages. Harnessing a corpus of lesser-documented languages makes it possible to persuasively suggest a universal phonetic effect of word-initial lengthening. The functional significance of this effect, namely for segmenting the continuous speech stream into a string of discrete meaningful units, is discussed in the context of extant literature on the topic. The manuscript thus provides an important key to our understanding of the formidable and fundamental task of finding linguistic structure in the acoustic tangle of speech. The paper is therefore of interest to linguists, speech scientists, psychologists and, given the possible universality of the phenomenon, anthropologists in general.

I have only a small number of relatively minor comments that the authors could take up in an updated version:

General question regarding the data: Some of the languages in this sample might have (lexical) stress, or even systematic word-initial stress. Is there a way to disentangle effects of stress on word-initial phones from pure word-initiality?

Unfortunately, our data are not annotated for supra-segmental features such as stress or tone. Therefore, we cannot systematically investigate prominence on the level of the word or the phrase. Based on the available sources on the 51 languages (and bear in mind that some of these languages are documented to a very limited degree), we compiled a table, added in the Supplementary Material B, that gives a general characterization of the word prosody systems found in the languages in our sample. We could find reliable descriptions of word prosody for 36/51 languages. Out of these 36 languages, 14 are

described as tone languages, 2 as phrase languages, and 20 as stress languages. Out of the 20 stress languages, 4 are characterized as having fixed initial stress, while the others are described as having fixed non-initial or non-fixed lexical stress. We do not observe any straightforward relation between the type of stress and the effect of word-initial lengthening. We have added this point to our discussion of limitations.

Line 074 et seqq

"Languages differ in their inventory and combinations of such acoustic cues [10], but one particularly widespread correlate for the prosodic organization of speech into different domains are the acoustic properties of word-initial phones [1, 11]."

The references to Cutler and Krivokapić do not really support the claim that the phenomenon under investigation (acoustic properties of word-initial phones) is "particularly widespread", since their investigations are mostly confined to English.

We agree and have rephrased the paragraph and changed the references. We have also made the languages for which this has been studied explicit in order to qualify the statement more explicitly (see comment for Reviewer 3).

Line 095 et seqq

"However, the reality and magnitude of initial lengthening is still debated, and previous findings could not always be reliably reproduced [17]."

The reference to Wightman et al. (1992) seems inaccurate, as this study wasn't concerned with initial lengthening but final lengthening.

We thank the reviewer for pointing this out. On page 1716, Wightman et al. (1992) write "We also see no evidence of a relationship between the word-initial consonant lengthening reported by Oller (1973) and the perceived size of the boundary". This is why we included a citation for Wightman et al. here. However, after re-reading the paper again carefully, it is not clear to us what this statement is based on, as the paper by Wightman et al. is concerned with final lengthening and does not discuss word-initial consonants at all, apart from the one sentence quoted above. For this reason, we replaced our previous statement by the following sentence: "However, very little is known about the extent and degree of word-initial lengthening across languages."

Line 101 et seq.

"In previous studies, utterance-initial consonants have been found to be lengthened or shortened, but with an overall small change in duration [18–20]."

Reading Amir et al. (2004), it appears that they look at initial syllables rather than consonants. Cruttenden (1997) discusses the phenomenon of "anacrusis", i.e. shortening of unstressed utterance-initial syllables. Since the authors refer

specifically to the segmental level (consonants), these references might not be fully appropriate.

We agree with the reviewer and have removed the inappropriate references.

Paragraph beginning in line 126

The purpose of this paragraph for the story presented here is not clear to me. The cohort model is not so much about word segmentation, rather about word identification, no? The asymmetry between word onset consonants and other consonants is more important for word segmentation than for word identification, I think.

We dealt with this comment in conjunction with Reviewer 2's comment regarding the first paragraph of the discussion and Reviewer 3's comments regarding the theoretical relevance of our study. In general, we decided to focus in the introduction on a review of various boundary phenomena reported in the literature so far, and omit reference to speech comprehension models like the cohort model. In the discussion, however, we made two basic theoretical assumptions more explicit: First, we explain how word identification and segmentation could interact in comprehension, including the role of incremental processing therein. And second, we make our assumptions about the (largely unclear) interaction between production and comprehension in this respect explicit. This, we hope, provides a clearer account of the causal relations we assume between acoustic lengthening and segmentation-advantages for listeners, without making any unwarranted claims.

"Consequently, wordinitial positions are assigned a prominent status in phonological theories [25–28]."

The word "consequently" is inappropriate here. Surely, the prominent status of word-initial consonants within phonological theories is not based on the cohort model, nor on speech processing more generally.

We agree and fixed this inappropriate wording during revision of this paragraph.

Statistical model – Figure 2, page 6

The factors "utterance-initial" and "word-initial position" are represented as "varying effects" (grey arrows). Looking at the model in the github repository, these factors were specified as fixed effects for which there are varying slopes in the random effects structure of the model. The other "varying effects" (grey arrows) were entered as random effects. I am not sure about the terminology applied here, but there seems to be a difference between "random effects" and "varying effects", or at least it seems that the grey arrows in Figure 2 conflate or confound two different types of effect in the model. Please clarify.

We see how the model definition as presented in the initial submission can be confusing. In general, there is no difference between 'random effects' and 'varying effects'. Both terms are used interchangeably in the statistical literature. Following Gelman (2005), we use the term 'varying effects'. He presents the following definition:

- We define effects (or coefficients) in a multilevel model as constant if they are identical for all groups in a population and varying if they are allowed to differ from group to group. (Gelman 2005, p. 21)

In the model, the effect is added both as a general term (a population level slope) as well as a varying slope (per-language variation of the effect). This is standard procedure for varying effects, since the varying slope is defined as varying in comparison to the population-level effect. We have added a short clarification to the model definition so that this becomes clearer.

I was curious to see that "sound class" was entered as random effect (explanation Line 720-727). The authors assume six different sound classes ((un)voiced plosive, (un)voiced fricative, (un)voiced sonorant). The difference between continuants (fricatives, sonorants) and non-continuants (plosives) could be construed as a fixed effect, since there is a relatively clear hypothesis that non-continuants are shorter than continuants. So at least this dimension would not properly be construed as "random" (I have no clear intuition/hypothesis regarding the durational distinction between fricatives and sonorants, or between voiced and unvoiced sounds).

In agreement with the comments of reviewer 1 and 2, we have re-structured our model with respect to the variability of lengthening effects between different segments. Instead of grouping the segments into six sound classes that only relate to the manner of articulation, we model the effect of word- and utterance-initial as potentially varying between all kinds of segments. This explicitly includes the manner *and* place of articulation. For example, velar nasals are allowed to have a different slope and intercept from alveolar stops, and velar stops have yet another slope and intercept. This fulfills the request from Reviewer 2 that 'phoneme-level distinctions should be modeled at the most finely-detailed level permitted by the data'. As the reviewer points out, there are intrinsic differences to the different places and manners of articulation with respect to segment duration. Statistically, this is justified through three important characteristics:

A) We have multiple observations for each kind of segment.
B) We have only a sample from the total population of segments: There is a set of phones that does not appear in our data.
C) We know that lengthening effects affect different phones differently.

To our surprise and satisfaction, the model has no problems with the identifiability of parameters, despite the 193 different levels to fit.

Line 496 et seqq.

"... that lengthening is more closely linked to the identification of word units than to prosodically structuring speech into larger units such as prosodic phrases."

Here, the authors might want to specifically refer to "initial lengthening", as it is known that final lengthening is definitely linked to prosodic structuring.

We agree and have changed it as suggested.

Line 539 et seqq.

"As lengthening can be one phonetic manifestation of phonological strength, this might suggest that we should find consistent differences in lengthening across sound classes."

The concept of "phonological strength" and its relation to length is not really clear to me. I could imagine that weakening might likewise lead to an increase in duration (think of lenition processes, such as spirantisation of underlying plosives).

A stylistic remark: the word "consistent" appears three times within this paragraph (four times on this page).

We have reconsidered the whole paragraph and decided to remove it, since it did not directly relate to our analysis and discussion.

Line 555 "be" --> "to"

final segments have been shown be undergo considerable lengthening

We have fixed this typo.

Line 553-563

Discussion about the differently expressed demarcative functions of initial and final lengthening.

There is another interesting parallel with final lengthening: Recent evidence suggests that, at very strong prosodic boundaries with long pauses, the final lengthening-effect vanishes (see Kentner et al. 2023), just as the word-initial lengthening effect is absent when the word commences an utterance. In both cases, this could be explained by an avoidance of redundant marking of boundaries (or lack of functional ambiguity due to a clear pause). [this study is part of my own work, please do not feel obliged to cite]

We thank the reviewer for this suggestion and agree that there is an interesting parallel between the findings in Kentner et al. (2023) and our own findings related to the avoidance of redundant marking. We have added a citation of the paper to the Discussion

section. We also make reference to their finding when discussing the implications of our results for the pi-gesture model (see below).

Discussion section

The results suggest that word-initial lengthening is just word-initial and not related to higher levels of prosodic structure. This finding thus affords good reasons to discuss a prominent model in articulatory phonology, namely the pi-gesture model. According to Byrd and Krivokapić (2021), this model assumes that final lengthening and initial lengthening are different phenomena that are due to a single process, namely the pi gesture, which somehow acts as a pacemaker, slowing down articulatory gestures around prosodic breaks; the stronger the break, the greater the slowdown.

Byrd and Krivokapić (2021: 37)

"Within the π-gesture model, boundaries are represented gradiently rather than categorically or symbolically. So, boundaries encoding greater disjuncture (for example, in traditional terms, at an Intonational Phrase versus an Intermediate Phrase) have greater activation and thereby exert a greater slowing or "stretching" of the concurrent gestural activations. The π-gesture model makes no distinction between final and initial phrase edges; a single π-gesture spans a boundary, reaching transgesturally into both final and initial phrase-edge material."

The authors of the present paper might want to respond to these considerations, as their results seem to be relevant in this context.

We have added a paragraph in the discussion on the pi-gesture. While the observation of word-initial lengthening fits the predictions of the model, this is not so much the case for the absence of additional lengthening effects for the utterance-initial position. There are several explanations for this, which we cannot fully pursue in the scope of this article, but some ideas are presented:

A) The pi-gesture lengthening and absence of lengthening for marking the boundary cancel each other out, and thus no additional lengthening compared to word-initial lengthening is observed.
B) The pi-gesture model needs modification, e.g. it might be that it does not reach transgesturally, and mainly operates as final-lengthening.

In any case, the predictions from the pi-gesture model in its strong reading do not hold up, since we do not observe more lengthening at utterance-initial compared to word-initial position. We relate this finding also to the observation of Kentner et al. (2023), who observe a similar (non-)effect for final lengthening.

Line 670 (typo "used / using")

We have fixed this typo.

References
Byrd, D., & Krivokapić, J. (2021). Cracking prosody in articulatory phonology. Annual review of linguistics, 7, 31-53.

Kentner, G., Franz, I., Knoop, C. A., & Menninghaus, W. (2023). The final lengthening of pre-boundary syllables turns into final shortening as boundary strength levels increase. Journal of Phonetics, 97, 101225.

Reviewer #2:

Remarks to the Author:

General comments

This is a worthwhile and original analysis of a wide range of languages which examines an important source of durational variation, one that is likely to be a segmentation cue for speakers across languages, and may indeed be universal (e.g., White, Benavides-Varela & Mády, 2020).

The data-sets are not well matched between languages, with samples ranging from 1 to 30 speakers and from less than 2000 words to more than 9000. This is not intrinsically a problem, as the key point here is not a comparison between languages, although cross-linguistic comparisons are reported (Figures 3 and 4). There are, thus, various limitations on the interpretations of observed cross-linguistic differences and the authors are rightly circumscribed about this, with a number of confounding factors noted in the discussion.

Sources of durational variation between languages may also relate to distributional factors. As discussed below, the authors need to more clearly account for: firstly, singleton consonants vs those in initial clusters (if these were distinguished, it was not clear from the methods); secondly, intrinsic durational variation due to place of articulation, which is not controlled here (see 720 point below).

It is a strength of the study that most languages analysed here are previously understudied as regards their durational properties. The inclusion of varieties of French and English does allow some checking of methods and analyses, however. There are many empirical studies of English speech timing, for example, and the magnitude of effects reported in the current paper for English (both word-initial and utterance-initial) could instructively be compared with those of earlier studies (for original sources, see reviews in, e.g., Fletcher, 2010; White, 2014).

We see that such a detailed comparison could be of interest; however, there are two main confounds that lead us to believe our paper is not the right place for such a discussion. First, our dataset contains observational data while previous studies on English and French were mainly concerned with experimental data, making a comparison difficult. Second, at least in the case of English, our data actually do not represent a mainstream variety of English but instead a dialect spoken in South(-Eastern) England, and we do not

see an *a priori* reason to assume that the exact details of how lengthening is manifested in that particular variety should be similar to those reported for, say, RP or American English. Therefore, we respectfully reject the offer to delve into this particular strand of discussion and focus on the cross-linguistic analysis instead.

Overall, this paper deserves to be widely read, given the implications for the understanding of phonetic universals. Such findings inform a wide range of theoretical domains, including language evolution and individual language development, and have obvious implications for language technologies.

Specific points are indexed by line numbers in the manuscript.

Introduction

para beginning 113: The discussion of potential phonotactic cues to word segmentation)“[language-] specific complex clusters”) is rather convoluted and could be shortened: some languages have effective consonant clustering cues and some do not.

We agree that this paragraph was a bit convoluted. We have reformulated the paragraph to make our line of argumentation easier to follow.

Methods

General: How were consonant clusters treated? These should definitely be analysed separately from singletons in onset position. Effects of coarticulation as well as variation in the distribution of prosodic lengthening effects (see 705 point below) mean that intrinsic durations in cluster onsets will tend to differ from those in singleton onsets. Such considerations (see also point 720 below) may well help to explain the observations of two languages that apparently lack word-initial lengthening in these data.

We thank the reviewer for this suggestion. The revised model now includes a variable ‘cluster_status’ that varies between languages. We have added three levels to this variable:

- Single consonant (no preceding or following consonant)
- Cluster-Initial
- Cluster-non-initial

On the population level, we find that cluster-initial consonants are longer than the others, and non-initial consonants are shorter. The effect for single consonants are in between these two categories. We also find that adding this control actually improves the model inference, but does not really affect the overall results. Together with the more detailed control for place and manner of articulation, the revised model can be considered as being more conservative.

682: Please could you give an indication of lower outlier thresholds? Were these, in practice, at 0ms or were they above that?

Accidentally, we had only included this threshold in the supplementary material, while it definitely should have been part of the main text. We thank the reviewer for pointing this out. We have added all necessary information for outlier filtering in the revised manuscript.

- We excluded sounds with a duration equal to or below 30ms, which was set as the minimum duration by the MAUS aligner, and smaller duration values are indicative of imprecise last-resort alignments.

The minimum value of 30ms leads to a pooling of all reduced/elided phones to that value. In case a corpus creator has annotated a phone because it `belongs there', but it's not actually spoken, it will receive the minimum value. If a phone is heavily reduced, the same applies. This means that all phones at this value are no reliable estimates, which is why we removed them from the dataset. This is irrespective of their position. Further details can be found in the methods article for building the DoReCo corpus (Paschen et al. 2020).

705: Note that "polysyllabic shortening" has been shown, at least for English, to be modulated by higher-level prosodic lengthening effects, i.e., the inverse relation between word length and the duration of sub-units (such as the primary stressed syllable) only holds when the word is subject to phrasal accent or final lengthening (e.g., White & Turk, 2010; White, 2014; also, e.g., Suomi, 2009, for Northern Finnish).

Thank you for the suggestion. We added a clarification and a reference to White (2014).

720: "we also control for variation of the effect across different sound classes, since there might be variation in the elasticity of segments depending on whether they are stops (and affricates), fricatives, or sonorants." There should also be control for place of articulation, as durational differences between bilabial, alveolar and velar stops (for example) are systematic and well-attested (e.g., Klatt, 1976). Moreover, differences are not simply due to variation in "elasticity", but also due to intrinsic articulatory constraints. As distribution of consonant types is not controlled between location (word-initial vs word-medial), and is moreover unlikely to be balanced (due to language-specific positional phonotactics), phoneme-level distinctions should be modelled at the most finely-detailed level permitted by the data.

In agreement with the comments of reviewer 1 and 2, we have re-modeled our model with respect to the control for sound class. Instead of grouping the segments into six sound classes that only relate to the manner of articulation, we model the effect of word- and utterance-initial as potentially varying between all kinds of segments. This explicitly includes the manner *and* place of articulation. For example, velar nasals are allowed to have a different slope and intercept from alveolar stops, and velar stops have yet another slope and intercept. This fulfills the request from Reviewer 2 that 'phoneme-level

distinctions should be modeled at the most finely-detailed level permitted by the data'. Statistically, this is justified through three important characteristics:

A) We have multiple observations for each kind of segment.
B) We have only a sample from the total population of segments: There is a set of phones that does not appear in our data.
C) We know that lengthening effects affect different phones differently (see Klatt 1976, as suggested by Reviewer 2).

To our surprise and satisfaction, the model has no problems with the identifiability of parameters, despite the 193 different levels to fit.

Results

221: "Varying slopes and intercepts have been included in a nested way for speakers and different sound classes for each language." Please clarifying. What is nested within what else?

Due to the model restructuring, this is no longer relevant. We have removed this sentence and explained our model structure in a less confusing way.

Discussion

General: The listener requirement for cues to both word segmentation and word recognition/lexical access, both of which are boosted by word-initial lengthening, is very likely to contribute to the apparent universality of word-initial lengthening (see White, Benavides-Varela & Mády, 2020, for extended discussion). That specific linkage - i.e., between segmentation and recognition - could be discussed at more depth here.

The reviewers partially disagree with respect to the discussion about speech segmentation and word identification. While reviewer 2 asks us to discuss this more in depth, reviewer 3 asks us to avoid any explicit claims about speech perception. In order to reconcile both comments, we have taken the following steps:

1. We have moved the discussion about potential (!) consequences of initial lengthening on speech segmentation and word identification to the discussion. Since our study analyzes lengthening in acoustic data and not in speech perception, the introduction mirrors this scope.
2. We elaborate on the potential role of word-initial lengthening for speech segmentation and word recognition more in depth in the discussion. In this context, we have also made a more explicit reference to White et al. (2020), who propose the universality of word-initial lengthening based on artificial language learning experiments with speakers of English, Italian, and Hungarian. However, we are explicit about the fact that we do not make claims about speech processing and perception directly. Rather, we explore the potential benefit of lengthened initial

consonants. The discussion is only about *potential* effects of lengthening for the hearer. Further experiments in the realm of speech perception will have to be carried out in order to make more precise claims about the cross-linguistic validity of such processes.

486-499 (also 108): The functional motivation for the lack of consistent utterance-initial lengthening was formulated earlier than the reference cited ([15], at line 108): thus, see White, 2002; White 2014.

We have adapted the reference to relate to the first formulation of the functional motivation, i.e. White (2002).

Typos
670: “using WebMAUS service was used”

We thank the reviewer for pointing this out and have fixed this typo.

Additional references

Klatt, D. H. (1976). Linguistic uses of segmental duration in English: Acoustic and perceptual evidence. The Journal of the Acoustical Society of America, 59(5), 1208-1221.

Suomi, K. (2009). Durational elasticity for accentual purposes in Northern Finnish. Journal of Phonetics, 37, 397-416.

White, L. (2002). English speech timing: A domain and locus approach. University of Edinburgh PhD dissertation.

White, L., & Turk, A.E. (2010). English words on the Procrustean bed: Polysyllabic shortening reconsidered. Journal of Phonetics, 38, 459-471.

Reviewer #3:

Remarks to the Author:

Summary: This article investigates word-initial lengthening, i.e. referring to the phenomenon when consonants at the beginning of a word are longer than in word medial and final position. The authors investigate this phenomenon in a sample of (semi)spontaneous speech across 51 languages. They report robust evidence for word-initial lengthening within their sample.

A unique strength of this paper is that it investigates a diverse (and for standards in the field large) sample of mostly non-WEIRD languages. Analyses such as the present one have the potential to provide valuable estimations of effect magnitude and variance across language (for the phenomenon under scrutiny). The authors

also make their scripts and data processing steps open and transparent for the reader to evaluate. I tip my head to the transparency demonstrated here.

Having said that, the paper has room for improvement: I suggest (1) toning down overconfident claims (or at least qualifying them), (2) spelling out the theoretical relevance of their findings, (3) discussing and taking into account possible dependencies between languages in their sample, and (4) presenting the evidence in a more appropriately conservative way.

We are very thankful for the considerate critique that the reviewer offers. Since we are committed to providing a paper that does not rely on overconfident claims, we are grateful for the detailed highlighting of where we had failed to do so in the initial submission. We take this matter seriously, and hope to be able to provide satisfying answers to the raised issues. We also welcome any further feedback in this direction.

Detailed comments below:

1. Overclaiming

The paper overconfidently presents evidence at times and I'd invite the authors to nuance their statements (or directly qualify them more often).

E.g. "one particularly widespread correlate for the prosodic organization of speech into different domains are the acoustic properties of word-initial phones" (ll. 76f)

Not only is the cross-linguitic coverage of this phenomenon narrow at best (and that is exactly why the present paper is a great contribution), but the evidence itself is also often based on experiments with only a few speakers. Please either nuance or qualify your statement.

We have made the languages for which such evidence exists explicit (at least English, French, Korean) and removed the reference to the previous studies as "particularly widespread" in favor of the more nuanced account of the existing literature.

E.g. "Despite these limitations, the robust patterns across a word-wide sample of languages suggest a statistically universal process of lengthening of word-initial consonants." (ll. 631f)

See below. I suggest to nuance this very strong claim the universality of the phenomenon in light of what we can actually learn from the data. I do not believe the paper needs to claim universality to be a valuable contribution.

In our original submission, we had Bickel's (2011) statistical universals in mind, a commonly used term in linguistic typology. However, we agree with the reviewer and have toned down our statement:

- "Despite these limitations, the evidence across a word-wide sample of languages suggest that lengthening of word-initial consonants is a potentially fundamental process structuring human speech."

2. Theoretical relevance

The paper leaves the assumed impact of the phenomenon vague, so I would like to invite the authors to make their claims more explicit.

E.g. "A compelling rationale for word-initial lengthening effects is the cohort model that describes the incremental processing of speech" (ll. 126f.). First, the cohort model does not describe but propose incremental processing mechanisms and it does so specifically for spoken word recognition. Second, the phrasing of this sentence leaves much room for interpretation. Do the authors propose that because humans seem to incrementally recognize words (i.e. as acoustic information comes in, lexical competitors compatible with the acoustic information are activated and incompatible ones are eliminated), acoustically marking word boundaries by durational cues helps that process? If so how exactly does initial lengthening affect this process? If there are other assumptions that the authors make, please specify the causal relationships you assume/propose. The authors continue two sentences later with "and asymmetries in the distributions of sounds play an intricate part in marking domain boundaries" (ll 133f). I do not fully understand this sentence: Asymmetries in the distribution of durational values? I recommend clarifying.

In considerations of comments from Reviewer 1 and Reviewer 3, we have re-modeled the relevant parts of the introduction. We no longer introduce the cohort model since it has no direct relation to our observation of acoustic data. We have also removed the other problematic parts identified by the reviewer and provide a more detailed introduction of the role of word onsets in general instead. With respect to the causal relationship between lengthening and spoken word recognition, see the next comment.

On lines 462f. the authors state "Our findings carry significant implications for understanding the incremental nature of lexical processing in natural human speech." I am puzzled by this statement. This paper does not show any link between initial lengthening and lexical processing. It is an assumption made by the authors (which also remains vague). The authors continue: "Longer consonantal segments at the onset of words may thus constitute one of the most fundamental strategies for the segmentation and identification of distinct linguistic units". This claim, again being very detached from the actual data presented, suggests that the authors actually mean that initial lengthening helps segmenting utterances into smaller units such as words. Albeit related, this is not the same as incremental processing in spoken word recognition as suggested in the introduction. I think the paper would benefit from nuancing these statements more

and making explicit what causal mechanisms are assumed here exactly, as well as how the data do or do not speak to these claims.

The reviewers partially disagree with respect to the discussion about speech segmentation and word identification. While reviewer 2 asks us to discuss this more in depth, reviewer 3 asks us to avoid any explicit claims about speech perception. In order to reconcile both comments, we have taken the following steps:

1. We have moved the discussion about potential (!) consequences of initial lengthening on speech segmentation and word identification to the discussion. Since our study analyzes lengthening in acoustic data and not in speech perception, the introduction mirrors this scope.
2. We elaborate on the potential role of word-initial lengthening for speech segmentation and word recognition more in depth in the discussion. In this context, we have also made a more explicit reference to White et al. (2020), who propose the universality of word-initial lengthening based on artificial language learning experiments with speakers of English, Italian, and Hungarian. However, we are explicit about the fact that we do not make claims about speech processing and perception directly. Rather, we explore the potential benefit of lengthened initial consonants. The discussion is only about *potential* effects of lengthening for the hearer. Further experiments in the realm of speech perception will have to be carried out in order to make more precise claims about the cross-linguistic validity of such processes.

While the assumption that prosodic cues help listeners to segment speech is not controversial, certain (rather small) acoustic asymmetries such as initial lengthening might also be mechanical consequences of how articulatory gestures are organised and coordinated. Whether the acoustic consequences of such assumed asymmetries are perceptually exploited across these languages remains an open question.

In agreement with comments from Reviewer 1 and 3, we have added a paragraph discussing the pi-gesture model that discusses lengthening of articulatory gestures at boundaries. Here, we also explore the idea that lengthening could be a *mechanical consequence* of this gesture coordination. However, we consider the absence of additional lengthening effects at utterance-initial position as evidence against a strict model of pi-gestures, since this would predict longer lengthening at utterance-initial than word-initial position.

3. Sampling

I would like to see more information and consideration of the sample. We know that languages are not independent data points due to areal and genetic relationships. This has been a common methodological bottle neck for using cross-linguistic data in inference. E.g three related languages showing the same property tell us

little about the generalisability of that property and should inferentially be taken into account (e.g. Roberts et al. 2015). I would like the paper to present (a) more information about possible dependencies between languages and (b) at least discuss the possible impact of these dependencies on their inferential procedure. Does the "robustness" of the pattern hold up when controlling for areal and genetic dependencies between languages? (but see my concerns about robustness below).

Roberts, S. G., Winters, J., & Chen, K. (2015). Future tense and economic decisions: Controlling for cultural evolution. PloS one, 10(7), e0132145.

The reviewer is right about pointing out the lack of control for areal and genetic dependencies in the first submission. We have fixed this with two additional computations. First, we have added varying intercepts for each language family. However, since we have more than 20 language families with only one language in the sample, the model does not assign a lot of variability to the variable language family. Second, we control for areal dependencies by computing Moran coefficients for the data of all macro areas in our sample. This coefficient measures the amount of spatial auto-correlation in the data. Since the languages in our sample that belong to the same language family (especially Austronesian, Indo-European, Sino-Tibetan) are also geographically close to each other, we interpret this as an additional approximation for controlling for phylogenetic biases in our data. For all macro areas, the Moran coefficient is close to 0, indicating no or very low spatial auto-correlation in the data.

4. Interpretation of evidence

The authors interpret the results of their Bayesian model as evidence for a robust pattern across languages.

I have several caveats with regard to this interpretation and the presentation of it. Note that I do believe the authors that we see a level of consistency that is worthwhile discussion, but I think it is worth being more humble when interpreting the results (which does not make the observation weaker at all, only more honest). I do not doubt that the data seem descriptively compelling and given the immense variability observed in spontaneous speech, that is sometimes as much as we can hope for, but the inferential presentation is in my opinion overconfidently presenting the results:

The inference solely relies on the interpretation of the population estimates and the estimated uncertainty around it. The credible intervals are interpreted relative to two reference values: point zero and the boundaries of a ROPE (which is not exactly defined in the paper as far as I can see, see below).

First, this form of inference is very dependent on the specified model, so generally not a very strong measure of evidence but it is not uncommon that researchers use this form of inference.

Second, the authors relate the reference values to the 80% HDI interval and claim that most languages' 80% HDIs are outside of said reference values. This is very conservative. Concretely it means for more than a few languages, a substantial amount of plausible predicted values cross the reference values. I recommend using a more conservative comparison with e.g. the 95% HDIs and nuance the authors' confidence.

We follow the suggestion of the reviewer and adopt a more conservative threshold than initially. Since we want to point out the arbitrariness of the exact number of the HPDI threshold for inference, we present our results with respect to two different thresholds: 89% and 95% (see McElreath 2020, p. 56). Both plot and text make these thresholds explicit. Furthermore, we present the results using posterior predictions on simulated data and report on the expected differences between segment positions.

With respect to the ROPE, please see our comment further below.

Third, I would like to see the ROPE clearly defined and theoretically justified. I was puzzled that on the one hand the authors mention an important perceptual threshold, the just noticeable difference which lends itself very well to be used as a ROPE, but apparently did not use that threshold as the ROPE (even though they do argue for the perceptual relevance of their effect magnitude).

The fact that the ROPE was not spelled out explicitly was a clear error and has been fixed with this revision.

As for using Quine's just-noticeable difference as threshold, we have several doubts which lead us to not use this as a general proposed threshold.

1) The threshold is, to the best of our knowledge, only tested with (Indo-)European languages. It is unclear whether the threshold would be lower (or higher!) for other languages with different phonological systems, tone, etc.
2) The threshold in Quine is computed against a 'base rate of speech' in an experimental setting of controlled utterances, which seems difficult to compare with our noisy observational data from spontaneous speech.
3) Our main comparison is not against a base rate of speech in the utterance (as in Quine), but against specific consonants in specific positions (e.g. word-medial, and utterance-initial). Thus, it is unclear how the 5% of difference actually relate to our data and model.
4) Considering the shrinkage that is inherent to the multilevel models (as the reviewer points out as well), the threshold postulated by Quine and the language-specific slopes in our model are not directly comparable.

We still think it is favorable to use a small ROPE instead of none (which would be the sad standard in quantitative linguistics), which is why we decided on the presented range of -0.01 and 0.01 on log-scale. For a segment of 100ms duration, this is about 1ms of difference. We do not know if a difference higher than that is actually noticeable (and we

will make no claims about this). But we are quite confident about the fact that a difference shorter than this is not.

The most relevant point is presented by the reviewer, in a position which we adapt. We do not have any analysis of speech processing – but Quine's threshold refers exclusively to speech perception. This does not relate one-to-one to acoustic data, and should not guide us here.

In line with the other comments, we have toned down the relation of our finding to the processing of speech. This includes qualifying the relevance of the 5% threshold. We do not want to claim that our effect is noticeable by listeners, since this is not what we are testing in our analysis, as rightfully pointed out by the reviewer in other comments. But then this threshold should also not lead us to design our statistical analysis (but it probably should in a study on speech processing).

Finally, the central figure 3 might be again overconfidently representing the nature of the data. The graph shows model estimates pulled from a hierarchical model, i.e. shrinkage will pull individual estimates towards the grand mean and the posterior distribution are much narrower than the empirical ones. I think readers would benefit more from a graph that plots (either additionally or separately) the raw data distributions in order to see if the data also suggest a unidirectional pattern.

In order to not solely base our inferences on the posterior distributions,, we present posterior simulations using the fitted model on simulated data. Those simulations shows that the model predicts longer consonants in word-initial positions. On average, the consonants in word-initial positions are 13ms longer than in other positions.

The raw distributions per position are already part of the supplementary material B (Figure 1). Since this does not control for any of the possible confounds, we prefer to not interpret too much into those figures. However, a mean difference of around ~6ms is observable even in the raw data.

In light of the omnipresent but healthy uncertainty in speech data, I suggest the authors revisit some statement in their discussion that are interpreting null results. Null results, particularly within an analysis using their inferential process, are not informative whatsoever, so I recommend rephrasing these statements.

E.g. ll 544f. "The effect is consistent across all sound classes, despite their differences in mode and articulation. The effect is also consistent between all speakers of our data, which points to a uniform effect beyond individual speakers and languages."

Both of these statements interpret the absence of "robust" effect estimates underlapping with 0 (or their ROPE) as evidence of consistency. They are merely

compatible with this interpretation but also not incompatible with alternative point estimates that are non-zero.

We have restructured the reporting of our results and payed special attention to not interpreting any null results. We also report the individual results for lengthening across sounds and speakers in the Supplementary material. For speakers, there is a strong trend for lengthening across the sample with only a few uninterpretable results. For individual segments, the results are mostly not interpretable.

5. Misc

"Clusters cannot be used as a universal method for the purpose of segmenting the beginning of a word..." (ll. 121f.) Do you mean clusters cannot be used as a domain of measurement?

Reference 24 is not the appropriate reference for its preceding sentence.

We have re-structured the relevant lines to be more precise about our argument. The main point is that since many languages lack consonant clusters all together, they cannot be used as the main cue for marking word onsets universally. Not directly related to the comment but to clusters in our data, it should be considered that we have now also added `cluster-status` as an additional variable of the model.

Neither figure 1a nor figure 1c have readable text elements. I recommend making those figures more accessible by increasing the font size

We have adapted two different solutions for this problem. Since a larger font on the world map of languages would lead to a non-recognizable map, we have removed the language labels and color-coded the points for language family instead. This should give some account for the phylogenetic diversity within our sample (31 different language families). For 1c, we have increased the font size of all small elements.

I recommend using a better contrast for the 80% HDI boxes in figure 3. The black median is difficult to see on the dark purple background.

We have adapted our color scheme to make Figure 3 more readable.

ll 522f. The term s-mobile is likely unfamiliar to many potential readers.

We agree, and we have removed the mention of this particular phenomenon. Instead, we are now citing the paper by Green (2003) for a general overview of deletion phenomena pertaining to extra-metrical consonants.

ll 557f. "However, evidence for final lengthening at the word level remains dubious at best". Please qualify this statement. Some people might say that evidence for

initial strengthening and lengthening is also dubious at best given the small and unjustified sample sizes in these experiments.

We have removed most of this paragraph due to the restructuring of the discussion. The essence of the paragraph (word-initial lengthening for word-boundaries, final lengthening for marking prosodic boundaries, e.g. at utterance-final position) has been added to our discussion of the pi-gesture model, which makes explicit prediction about lengthening effects at different prosodic boundaries.

The authors note that they use word frequency as a covariate (ll 695f.). Where did they take these frequencies from?

We have added a short sentence that explains that the word-form frequency is computed as the frequency of each form within the DoReCo corpus of each language. While the corpora are quite small, they form the only source for such a calculation for many of the languages involved.

The authors note that their covariates are highly correlated. Correlated predictors lead to collinearity which can be a problem when interpreting coefficients. (e.g. Tomaschek et al. 2018). How did the authors tackle this potential bias?

We only observed such high correlation between the word-form frequency and the phonemes-per-word variables. This correlation is a known linguistic phenomena (more frequent words become shorter over time) and is thus not surprising. We have added a short explanation that makes it explicit that the parameter values of those effects should not be interpreted in isolation, but rather always be considered together. But since our main focus lies in the effect of segment position, we do not actually interpret the effect of these parameters and limit ourselves to a short mention of their posterior distribution and collinearity.

ll 676f.: "We also removed non-pulmonic consonants such as clicks, implosives and ejectives due to the large differences in articulation, compared to pulmonic consonants." I must have missed something, because I do not understand the justification for removing these consonants. Them being different should not affect the relative lengthening of them in certain phonotactic positions.

We have discussed the exclusion of these consonants again and decided to follow the suggestion of the reviewer. In the revised models, those consonants are no longer excluded. Instead, we control for the variation of lengthening effects by letting them vary per all segments (see comments on R1 and R2 on place/manner of articulation).

ll 682f. "Lastly, we excluded outliers beyond three standard deviations of the mean for each speaker." This decision artificially reduces variance. Can the authors

justify this choices? Even in a perfect normal distribution, 0.3% of data fall outside of 3 SDs.

Previous to the initial submission, we had made a random sample of excluded sounds and verified that those are indeed cases that should be excluded. Examples are erroneous alignments at the word-level, or transcription errors in the original corpus. Given the size of the corpus, those cases cannot be avoided right now, but will hopefully be excluded in upcoming versions of the database. We expand on this in the article. Further details can be found in the methods article for building the DoReCo corpus (Paschen et al. 2020).

Refs:
Tomaschek, F., Hendrix, P., & Baayen, R. H. (2018). Strategies for addressing collinearity in multivariate linguistic data. Journal of Phonetics, 71, 249-267.

References

Baayen, R.H., & Milin, P. (2010). Analyzing reaction times. *International Journal of Psychological Research*, 3(2), 12-28. http://dx.doi.org/10.21500/20112084.807

Bickel, B. (2011). "Statistical modeling of language universals" *Linguistic Typology* , vol. 15, no. 2, pp. 401-413. https://doi.org/10.1515/lity.2011.027

Gelman, A. (2005). "Analysis of variance—why it is more important than ever." *The Annals of Statistics* 33 (1) 1 - 53. https://doi.org/10.1214/009053604000001048

McElreath, R. (2020). *Statistical Rethinking: A Bayesian Course with Examples in R and Stan*. Chapman and Hall/CRC. https://doi.org/10.1201/9780429029608

Paschen, L., Delafontaine, F., Draxler, C., Fuchs, S., Stave, M., & Seifart, F. (2020, May). Building a time-aligned cross-linguistic reference corpus from language documentation data (DoReCo). In *Proceedings of the Twelfth Language Resources and Evaluation Conference* (pp. 2657-2666). https://aclanthology.org/2020.lrec-1.324/

Winter, B. (2019). *Statistics for Linguists: An Introduction Using R*. Routledge. https://doi.org/10.4324/9781315165547

Decision Letter, first revision:

Dear Dr. Blum,

Thank you for your patience as we've prepared the guidelines for final submission of your Nature Human Behaviour manuscript, "Consonant lengthening marks the beginning of words across a diverse sample of languages" (NATHUMBEHAV-23124306A). Please carefully follow the step-by-step instructions provided in the attached file, and add a response in each row of the table to indicate the

changes that you have made. Please also check and comment on any additional marked-up edits we have proposed within the text. Ensuring that each point is addressed will help to ensure that your revised manuscript can be swiftly handed over to our production team.

We would hope to receive your revised paper, with all of the requested files and forms within two-three weeks. Please get in contact with us if you anticipate delays.

When you upload your final materials, please include a point-by-point response to any remaining reviewer comments.

If you have not done so already, please alert us to any related manuscripts from your group that are under consideration or in press at other journals, or are being written up for submission to other journals (see: https://www.nature.com/nature-research/editorial-policies/plagiarism#policy-on-duplicate-publication for details).

Nature Human Behaviour offers a Transparent Peer Review option for new original research manuscripts submitted after December 1st, 2019. As part of this initiative, we encourage our authors to support increased transparency into the peer review process by agreeing to have the reviewer comments, author rebuttal letters, and editorial decision letters published as a Supplementary item. When you submit your final files please clearly state in your cover letter whether or not you would like to participate in this initiative. Please note that failure to state your preference will result in delays in accepting your manuscript for publication.

In recognition of the time and expertise our reviewers provide to Nature Human Behaviour's editorial process, we would like to formally acknowledge their contribution to the external peer review of your manuscript entitled "Consonant lengthening marks the beginning of words across a diverse sample of languages". For those reviewers who give their assent, we will be publishing their names alongside the published article.

Cover suggestions

We welcome submissions of artwork for consideration for our cover. For more information, please see our guide for cover artwork.

If your image is selected, we may also use it on the journal website as a banner image, and may need to make artistic alterations to fit our journal style.

Please submit your suggestions, clearly labeled, along with your final files. We'll be in touch if more information is needed.

ORCID

Non-corresponding authors do not have to link their ORCIDs but are encouraged to do so. Please note that it will not be possible to add/modify ORCIDs at proof. Thus, please let your co-authors know that if they wish to have their ORCID added to the paper they must follow the procedure described in the following link prior to acceptance: https://www.springernature.com/gp/researchers/orcid/orcid-for-nature-research

Nature Human Behaviour has now transitioned to a unified Rights Collection system which will allow our Author Services team to quickly and easily collect the rights and permissions required to publish your work. Approximately 10 days after your paper is formally accepted, you will receive an email in providing you with a link to complete the grant of rights. If your paper is eligible for Open Access, our Author Services team will also be in touch regarding any additional information that may be required to arrange payment for your article.

Please note that *Nature Human Behaviour* is a Transformative Journal (TJ). Authors may publish their research with us through the traditional subscription access route or make their paper immediately open access through payment of an article-processing charge (APC). Authors will not be required to make a final decision about access to their article until it has been accepted. Find out more about Transformative Journals

Authors may need to take specific actions to achieve compliance with funder and institutional open access mandates. If your research is supported by a funder that requires immediate open access (e.g. according to Plan S principles) then you should select the gold OA route, and we will direct you to the compliant route where possible. For authors selecting the subscription publication route, the journal's standard licensing terms will need to be accepted, including self-archiving policies. Those licensing terms will supersede any other terms that the author or any third party may assert apply to any version of the manuscript.

For information regarding our different publishing models please see our Transformative Journals page. If you have any questions about costs, Open Access requirements, or our legal forms, please contact ASJournals@springernature.com.

Please use the following link for uploading these materials:
[REDACTED]

If you have any further questions, please feel free to contact me.

Best regards,
[REDACTED]

On behalf of

[REDACTED]

Reviewer #1:
Remarks to the Author:
The authors have answered all comments in a satisfactory manner. Apart from one typo and a small remark regarding a Figure caption (see below), I have no further concerns, the paper can now be published.

line 616: "could potentially explained" --> "be" is missing

Caption of Fig 4: the term "utterance initial lengthening" is confusing, as the highlighted HPDI-boxes show shortening rather than lengthening. The authors should use a more neutral wording here (phone duration?).

Reviewer #2:
Remarks to the Author:
The authors have been assiduous in responding to the reviewers' comments. The study remains a valuable additional to the literature on phonetic universals. I have just a few further observations regarding points of clarification.

181: "Indeed, from a functional perspective, it makes sense that no additional cue to word segmentation is necessary at the beginning of utterances…" (also 542 etc. in the discussion): You mentioned in the rebuttal document that the citation had been amended to the earliest formulation of this interpretation of the lack of utterance-initial lengthening (White 2002 – also White 2014), but this was not the case in the revised manuscript. This is indeed my work, but I am sure you can appreciate my preference for being appropriately cited on such a specific point.

354: "There is a strong correlation ($\rho = 0.61$) between both parameters, in that many phones per word correlates with a lower word-form frequency […] Given the strong correlation between both

parameters, the effects should not be interpreted separately, but always be considered together." This is unclear. These effects are essentially independent. More frequent words do indeed tend to have fewer syllables. Additionally, in words with the same number of syllables, the segments in those of higher frequency may tend to be shorter (for various reasons). The combination of these effects would (perhaps marginally) tend to cancel out the differential impact of so-called "polysyllabic shortening" (see my previous review regarding how that can be reinterpreted, as now mentioned in the manuscript): thus, there are distinct shortening pressures on segments in many words of more syllables (loosely "polysyllabic shortening") and some words of fewer syllables (frequency effects). Some rewriting would be helpful here to deconfound these issues.

420 and elsewhere: "spatial auto-correlation" or "spatial autocorrelation" -> Be consistent with hyphenation.

757: "We excluded sounds with a duration equal to or below 30ms, which was set as the minimum duration by the MAUS aligner, with shorter durations being indicative of imprecise last-resort alignments…" It would be worth briefly noting that this exclusion may have some consequences for observed durational patterns, as in some cases, consonants may actually have very short durations (not simply due to measurement error). This is particularly likely utterance-initially: at least for English, utterance-initial nasals, for example, can be realised with very few pitch periods (reviewer's personal observation – citations may be available). Moreover, utterance-initial voiceless stops will only be acoustically evidenced by the stop-release phase, the preceding closure being unmeasurable (e.g., Turk et al., 2006); moreover, voiced stops are often devoiced utterance-initially (e.g., Solé, 2018 – which also discussing some constraints on articulation that may be relevant to the observation of utterance-initial shortening).

The year is missing for reference 86 (Piantadosi et al.).

Additional references

Solé, M.-J. (2018). Articulatory adjustments in initial voiced stops in Spanish, French and English, Journal of Phonetics, 66, 217-241.

Turk, A., Nakai, S. & Sugahara, M. (2006). Acoustic Segment Durations in Prosodic Research: A Practical Guide. In Methods in Empirical Prosody Research, edited by Stefan Sudhoff, Denisa Lenertova, Roland Meyer, Sandra Pappert, Petra Augurzky, Ina Mleinek, Nicole Richter and Johannes Schließer, Berlin, Boston: De Gruyter (pp. 1-28).

Reviewer #3:
Remarks to the Author:
I reviewed the first submission of the article (reviewer #3). The reviewer self-identifies as Timo Roettger (timo.b.roettger@gmail.com), in case the authors have any questions about my earlier comment or the ones that I am about to make. Overall, I am happy to see that the authors have revised their manuscript according to the useful suggestions made by the other two reviewers. They also seriously engaged with my own comments. The resulting revised manuscript is, what I consider, a much stronger paper. I have some rather minor comments about (a) certain formulations and (b) the visualisations. Maybe these are useful.

ll. 271ff: Here and later the authors use the term "evidence", reflecting their choice of interpreting the "underlap" of HPDI and reference values. Maybe it is worth to tell the reader at this first mentioning that his is your assumption / choice. So basically one could front the information in the next sentence as in "We interpret 89% HPDIs not overlapping with zero as evidence for…" or something along these lines. Then the authors could tell the reader that there is "evidence" for an effect.

ll. 280ff: "This is a measure to exclude spurious effects and to produce reliable results." This sentence strikes me as rather empty. If the authors bring it up, the reader could benefit from a short explanation how this helps avoiding spurious effects and produce reliable results. I, for one, am not convinced this is by default true. If I use a ROPE that is tiny, inferences based on a point zero reference are not that different from a ROPE approach. So I'd appreciate a short explanation here (plus a reference to a relevant study showing its benefits).

ll 290ff: "although this threshold has only been reported for specific kinds of experimental data so far." Without knowing of the exchange between reviewers and authors, I would be confused by this hedge here. Why bring up the JND if you do not find it a useful threshold? I suggest either discuss it (if you think this is useful, maybe in a footnote) or don't mention it. The hedge might confuse the reader.

l. 336: "Results at the population level". This header is confusing to me. The previous section already discussed population level results (in the technical sense) because the authors interpret the population level effect of position. Maybe the authors could make reference here to the control variables?

In this section, I also recommend including the HDPI range for all "effects", so that the reader can immediately interpret the uncertainty associated with them.

ll. 360ff.: "Given the strong correlation…". Would it be useful to direct the reader to a relevant reference on collinearity?

ll. 456f.: "… the distribution of the effect indicates a universal tendency in spoken language". I

understand the sexappeal of the word “universal” but it strikes me as heavily loaded. I understand that the authors already soften it by using “tendency”, but maybe “generalisable” is a more nuanced and less loaded way of saying the same thing.

ll. 529f.: “which can be explained by avoidance of redundant marking of boundaries.” I am a bit sceptical about that line of argumentation. Languages do not avoid redundancy at all. Redundancy is literally everywhere in language and it has been argued to make the communication system robust against perturbation and noise. See for example Winter, B. (2014). Spoken language achieves robustness and evolvability by exploiting degeneracy and neutrality. BioEssays, 36(10), 960-967. (Winter calls it “degeneracy” here).

Figure 5: I am not sure this plot is very useful. At least the intended visualisations of the posterior distributions are barely visible due to the scale. The authors could merely plot the posterior estimates and the HDPIs as whiskers to avoid this issue.

Figure 6: This is the plot that is supposed to, on a glance, show the main findings of the study. But I am not sure this is achieved here. The distributions are heavily overlapping, so it is hard to see differences in mean values. Yes the word-initial category is slightly shifted up, but it is not very apparent. Moreover, since these are posterior draws, the distributional information is redundant and uninformative because the priors of the model determined the shape of that distribution (not the data). I recommend a simple point and whiskers plot that plots estimates and the HDPI. That way, the reader can quickly see the shifted values for the word-initial category alongside the model uncertainty.

Also on a side-note: the y-axis is not log as stated, is it? And now that I look at it, the model was not informed that values below 30ms are not possible, was it (through priors)? I don’t think this would change anything, it’s just a mismatch between data and model assumptions.

In the hope some of these comments are useful.
Timo Roettger (timo.b.roettger@gmail.com)

Final Decision Letter:

Dear Mr Blum,

We are pleased to inform you that your Article "Consonant lengthening marks the beginning of words across a diverse sample of languages", has now been accepted for publication in Nature Human Behaviour.

Please note that *Nature Human Behaviour* is a Transformative Journal (TJ). Authors may publish their research with us through the traditional subscription access route or make their paper immediately open access through payment of an article-processing charge (APC). Authors will not be required to make a final decision about access to their article until it has been accepted. Find out more about Transformative Journals

Authors may need to take specific actions to achieve compliance with funder and institutional open access mandates. If your research is supported by a funder that requires immediate open access (e.g. according to Plan S principles) then you should select the gold OA route, and we will direct you to the compliant route where possible. For authors selecting the subscription publication route, the journal's standard licensing terms will need to be accepted, including self-archiving policies. Those licensing terms will supersede any other terms that the author or any third party may assert apply to any version of the manuscript.

Once your manuscript is typeset and you have completed the appropriate grant of rights, you will receive a link to your electronic proof via email with a request to make any corrections within 48 hours. If, when you receive your proof, you cannot meet this deadline, please inform us at rjsproduction@springernature.com immediately. Once your paper has been scheduled for online publication, the Nature press office will be in touch to confirm the details.

Acceptance of your manuscript is conditional on all authors' agreement with our publication policies (see http://www.nature.com/nathumbehav/info/gta). In particular your manuscript must not be published elsewhere and there must be no announcement of the work to any media outlet until the publication date (the day on which it is uploaded onto our web site).

If you have posted a preprint on any preprint server, please ensure that the preprint details are updated with a publication reference, including the DOI and a URL to the published version of the article on the journal website.

An online order form for reprints of your paper is available at https://www.nature.com/reprints/author-reprints.html. All co-authors, authors' institutions and authors' funding agencies can order reprints using the form appropriate to their geographical region.

We welcome the submission of potential cover material (including a short caption of around 40 words) related to your manuscript; suggestions should be sent to Nature Human Behaviour as electronic files (the image should be 300 dpi at 210 x 297 mm in either TIFF or JPEG format). Please note that such pictures should be selected more for their aesthetic appeal than for their scientific content, and that colour images work better than black and white or grayscale images. Please do not try to design a cover with the Nature Human Behaviour logo etc., and please do not submit composites of images related to

your work. I am sure you will understand that we cannot make any promise as to whether any of your suggestions might be selected for the cover of the journal.

You can now use a single sign-on for all your accounts, view the status of all your manuscript submissions and reviews, access usage statistics for your published articles and download a record of your refereeing activity for the Nature journals.

To assist our authors in disseminating their research to the broader community, our SharedIt initiative provides you with a unique shareable link that will allow anyone (with or without a subscription) to read the published article. Recipients of the link with a subscription will also be able to download and print the PDF.

As soon as your article is published, you will receive an automated email with your shareable link.

In approximately 10 business days you will receive an email with a link to choose the appropriate publishing options for your paper and our Author Services team will be in touch regarding any additional information that may be required.

If you have any questions about our publishing options, costs, Open Access requirements, or our legal forms, please contact ASJournals@springernature.com

We look forward to publishing your paper.

With best regards,
[REDACTED]
